# Altitude‐Associated Divergence of the Gut Microbiome in Endangered Forest Musk Deer: Evidence From Integrated Metagenomics, Metabolomics, and Culturomics

**DOI:** 10.1111/eva.70285

**Published:** 2026-06-22

**Authors:** Feiyun Huang, Zexiu Zhang, Yanni Zhao, Sen Ye, Maoyuan Gan, Xuxin Li, Yele Zhang, Lei Chen, Yushuo Zhang, Lingxi Chen, Ting Wang, Jinming Huang, Xiuyue Zhang

**Affiliations:** ^1^ Key Laboratory of Bio‐Resources and Eco‐Environment (Ministry of Education), College of Life Sciences Sichuan University Chengdu China; ^2^ Zhangzhou Pien Tze Huang Pharmaceutical Co., Ltd. Zhangzhou China; ^3^ Huang Jinming National Veteran Pharmacist Inheritance Studio Zhangzhou China; ^4^ The Conservation of Endangered Wildlife Key Laboratory of Sichuan Province, College of Life Sciences Sichuan University Chengdu China

**Keywords:** conservation metagenomics, environmental filtering, forest musk deer, gut microbiome, high altitude, short‐chain fatty acid

## Abstract

High‐altitude environments expose mammals and their gut symbionts to multifaceted stressors—hypoxia, cold, and intense UV radiation. Whether gut microbial communities undergo compositional restructuring in response to these stressors, and whether such restructuring carries translational value for captive conservation, remain unresolved questions. Here, we integrated deep shotgun metagenomics (≥ 15 Gb per sample), untargeted fecal metabolomics, and culturomics in 75 captive forest musk deer ( *Moschus berezovskii* Flerov, 1929) housed at high altitude (~3900 m) and low altitude (~1450 m) facilities under uniform husbandry. Neutral community modeling showed a greater contribution of deterministic processes at high altitude (only 34.3% of species conformed to neutral expectations vs. 89.3% at low altitude), consistent with stronger environmental filtering. At high altitude, we observed enrichment of a functionally coherent guild of short‐chain fatty acid (SCFA)‐producing bacteria—centered on *Flavonifractor plautii* , *Intestinimonas butyriciproducens*, and *Enterococcus faecium* —that formed antagonistic co‐occurrence networks with opportunistic pathogens including *Clostridioides difficile* and *Campylobacter* species, mirroring SCFA enrichment in phylogenetically diverse high‐altitude mammals. Fecal metabolomics revealed coordinated shifts in urolithin biosynthesis, branch‐specific regulation of the tryptophan–kynurenine pathway, and energy metabolism remodeling, all robustly predicted by microbiome composition via neural network modeling. Culturomics yielded seven safety‐validated isolates with confirmed gastrointestinal stress tolerance and broad‐spectrum pathogen‐antagonistic activity in vitro. These findings provide an actionable framework for altitude‐informed facility siting, fecal microbiota transplantation (FMT) donor selection, host‐derived probiotic development, and non‐invasive health surveillance in captive endangered species, and are broadly transferable to other taxa facing microbiome‐associated disease pressure in captivity.

## 1 Introduction

Ex situ conservation has rescued several flagship species from the brink of extinction (Conde et al. 2011; Snyder et al. 1996), yet a persistent paradox remains: certain species exhibit higher disease incidence and mortality in captivity than in the wild (Clubb and Mason 2003; Trevelline et al. 2019). The forest musk deer ( *Moschus berezovskii* Flerov, 1929) epitomizes this paradox in an especially acute form. The species is listed as Endangered by the IUCN (Wang and Harris 2015), and its wild populations have declined by more than 97% over the past seven decades (Yang et al. 2003; Jiang et al. 2023)—a collapse that makes captive breeding indispensable rather than auxiliary. Within captive forest musk deer herds, the same general pattern reported across other captive ungulates is dramatically amplified: chronic mortality from gastrointestinal disorders and bacterial infections—most prominently suppurative disease driven by *Trueperella pyogenes* —persists even under standardized management (Jiang et al. 2021; Zhao et al. 2011), establishing this species as a paradigmatic case in which the captivity‐associated disease burden directly threatens species survival. Against this backdrop, a fundamental question arises: which environmental variables most strongly associate with the assembly of host‐associated microbial communities, and how do the resulting configurations shape downstream health trajectories and, ultimately, conservation outcomes (Trevelline et al. 2019)?

The intestinal microbiome is central to nutrient assimilation, immune maturation, and colonization resistance (Sonnenburg and Bäckhed 2016; Lynch and Pedersen 2016), and its disruption has been repeatedly linked to disease vulnerability (Franzosa et al. 2019; Gilbert et al. 2018). Microbial communities are not randomly assembled: they are shaped by the interplay between deterministic ecological selection (environmental filtering, interspecific interactions) and stochastic processes (dispersal limitation, ecological drift) (Sloan et al. 2006; Ning et al. 2019). Among the environmental variables that could impose differential selection on captive microbiomes, altitudinal gradients offer a particularly tractable natural experimental system. Wild forest musk deer inhabit montane forests at 2500–4000 m (Yang et al. 2003), where they face the same chronic hypoxia, cold, and intense UV exposure that have driven convergent genomic adaptations in other high‐altitude mammals (Qiu et al. 2012; Ge et al. 2013; Wu et al. 2020). These host‐level physiological adaptations also reshape the intestinal biochemical milieu in which the gut microbiome assembles, raising the possibility that the gut microbiome of high‐altitude mammals is itself shaped by altitude‐associated environmental pressures (Storz et al. 2010). Consistent with this view, recent comparative studies have documented convergent increases in SCFA‐producing bacteria, particularly *Lachnospiraceae* and *Ruminococcaceae*, in Tibetan humans, Tibetan pigs, yaks, and macaques (Zhao et al. 2024; Ma et al. 2025; Zhang et al. 2016; Zeng et al. 2020; Li et al. 2023). Because butyrate sustains colonocyte energy balance under hypoxia through hypoxia‐inducible factor (HIF) signaling (Kelly et al. 2015) and confers systemic anti‐inflammatory benefits, any analogous enrichment in captive forest musk deer would carry direct implications for altitude‐informed management. Multiple captive breeding facilities along this altitudinal gradient under standardized husbandry constitute a tractable empirical setting for testing these ecological predictions.

Prior microbiome surveys of musk deer have relied largely on 16S rRNA amplicon sequencing and have documented captivity‐associated dysbiosis (Hu et al. 2017; Jiang et al. 2022, 2023), but none has treated altitude as a primary environmental predictor or corroborated compositional differences with metabolomic data. To address this gap, we systematically tested four interrelated hypotheses: (i) high‐altitude environments are associated with a greater contribution of deterministic processes to gut community assembly; (ii) this environmental filtering enriches a functionally coherent guild of SCFA‐producing bacteria while competitively excluding opportunistic pathogens; (iii) these taxonomic shifts translate into detectable functional consequences at the fecal metabolome level; and (iv) specific microbe–metabolite associations underlie the health‐protective potential of the high‐altitude microbiome. Together, these hypotheses predict that the high‐altitude gut microbiome constitutes a functionally structured community co‐shaped by altitude‐associated environmental pressures—a prediction with direct implications for altitude‐informed captive management. To test these hypotheses across two captive populations at contrasting altitudes (Tibet, ~3900 m vs. Qinling, ~1450 m; *n* = 75), we adopt an integrative three‐tiered framework: *pattern discovery* via deep shotgun metagenomics, *mechanistic parallel* via untargeted metabolomics interrogated jointly with metagenomic data through machine‐learning‐based microbe–metabolite association analysis, and *translational bridge* via culturomics‐based recovery of cultivable, functionally relevant bacterial isolates that sequencing and metabolomics alone cannot supply.

## 2 Materials and Methods

### 2.1 Study Sites and Sample Collection

We obtained fecal samples from two geographically separated captive forest musk deer ( *M. berezovskii* ) breeding stations situated at markedly different elevations within China: the high‐altitude facility in the Tibet Autonomous Region (31.23° N, 94.36° E; ~3900 m; mean annual temperature 3.5°C; plateau sub‐frigid monsoon climate) and the low‐altitude facility in the Qinling Mountains, Shaanxi Province (34.28° N, 106.78° E; ~1450 m; mean annual temperature 12.8°C; temperate continental monsoon climate). Both facilities employed standardized management protocols, with animals housed individually and provided daily with quantified concentrate supplements, fresh leaves, and drinking water.

Fresh fecal samples (*n* = 75) were collected between June and October 2024, within a single calendar year to avoid inter‐annual confounding. All individuals underwent standardized pre‐sampling health assessment—on‐site veterinary examination, body‐condition scoring, rectal temperature measurement, and 72‐h retrospective review of husbandry records—and only individuals without clinical signs of illness were included. We acknowledge that symptom‐based screening cannot rule out asymptomatic carrier states for subclinical enteric pathogens, and this limitation is noted in Section 4.7. To minimize contamination, the central portion (5–8 g) of each fecal deposit was collected within 5 min of natural defecation using sterile spatulas and a double‐glove protocol, transferred to pre‐labeled DNase/RNase‐free cryogenic vials, flash‐frozen in liquid nitrogen, delivered to the laboratory within 2 h, and maintained at −80°C pending extraction.

The sample set comprised 33 individuals from the high‐altitude facility (24 males, 9 females; 12 subadults, 21 adults; 20 summer, 13 autumn samples) and 42 from the low‐altitude facility (20 males, 22 females; 14 subadults, 28 adults; 21 summer, 21 autumn samples). Subadults were defined as sexually immature individuals (typically < 2 years) and adults as sexually mature individuals (typically ≥ 2 years).

### 2.2 Genomic DNA Isolation, Library Preparation, and Shotgun Metagenomic Sequencing

Whole‐community genomic DNA was isolated with the QIAamp PowerFecal Pro DNA Kit (Qiagen) following the manufacturer's instructions, incorporating mechanical bead‐beating for uniform lysis. Yield was measured on a Qubit 4.0 fluorometer; integrity was confirmed on 1% agarose gels. Shotgun libraries were prepared from 0.2 μg input DNA, with acoustic shearing to ~350 bp insert size (Covaris M220), and amplified for 8 PCR cycles. Final libraries were quantified by Qubit and size‐profiled on an Agilent 2100 Bioanalyzer. Paired‐end reads (2 × 150 bp) were generated on the Illumina NovaSeq 6000 platform at Novogene Co. Ltd. (Beijing, China), with a minimum depth of 15 Gb per sample.

### 2.3 Quality Control and Host Genome Filtering

Raw reads were filtered with fastp v0.23.4 (Chen et al. 2018); only reads ≥ 100 bp with mean Phred score ≥ Q20 were retained, and reads harboring adapter contamination or > 10% ambiguous bases were discarded. Host‐derived sequences were depleted by mapping the filtered reads to the forest musk deer reference assembly (GenBank: GCA_022376915.1; scaffold‐level, ~2.8 Gb, scaffold N50 ~102.4 Mb, BUSCO completeness 97.1% against cetartiodactyla_odb10) via KneadData v0.12.0 coupled with Bowtie2 v2.5.1 (Langmead and Salzberg 2012). Per‐sample read processing statistics are provided in Table S1.

### 2.4 Taxonomic and Functional Profiling

Reads were taxonomically assigned with Kraken2 v2.1.3 (Wood et al. 2019) against the PlusPF index (Lu et al. 2022), with abundance re‐estimation by Bracken v2.8 (Lu et al. 2017); relative abundances were derived by dividing each taxon's reads by the total classified read count per sample. Community functional potential was inferred using HUMAnN3 v3.7 (Beghini et al. 2021) against the ChocoPhlAn pangenome (99,200 genomes from 5989 species) and UniRef90. Species‐stratified pathway abundances were expressed as copies per million (CPM). Metabolic pathways from MetaCyc (Caspi et al. 2020) and carbohydrate‐degrading enzyme families from CAZy (Drula et al. 2022) were annotated to characterize the degradative and biosynthetic repertoire.

### 2.5 Community Diversity, Assembly, and Network Analyses

Genus‐level alpha diversity (Shannon, Simpson, Pielou evenness) was compared by Mann–Whitney *U* tests after Shapiro–Wilk tests confirmed non‐normality. For beta diversity, Bray–Curtis dissimilarity matrices were ordinated by PCoA, and compositional divergence was evaluated by PERMANOVA (999 permutations; vegan R package) with altitude, sex, age, and season as predictors.

Two complementary ecological null models were employed. First, the Sloan neutral community model (NCM; Sloan et al. 2006; Burns et al. 2016) was fitted at the genus level using the “spaa” and “Hmisc” R packages. For each genus, the observed occurrence frequency (proportion of samples in which it was detected at non‐zero relative abundance) was related to its mean relative abundance across the metacommunity through β‐binomial regression, fitted by a single parameter (the migration rate *m*); the 95% binomial confidence interval around the fitted prediction curve defined the neutrality envelope within which a genus was classified as conforming to neutral expectations, whereas genera positioned above (occurring more frequently than predicted) or below (less frequently than predicted) the envelope were classified as subject to positive or negative selection, respectively. Second, the normalized stochasticity ratio (NST) was computed on Ruzicka dissimilarity matrices (Ning et al. 2019); values below 50% indicate deterministic dominance. The modified stochasticity ratio (MST) was computed as an additional metric. Together, these capture both taxon‐specific selective pressures (NCM) and the community‐wide balance of assembly mechanisms (NST/MST).

Species‐level co‐occurrence networks were constructed independently for each altitude group. Taxa present in < 20% of samples were excluded; only pairwise associations with |Spearman *ρ*| > 0.6 and FDR < 0.01 were retained. Network topology (node/edge counts, clustering coefficient, modularity) was characterized, and robustness was quantified as the proportion of nodes remaining in the largest connected component at 50% node removal, assessed through sequential deletion simulations under both random and targeted attack scenarios. Because stringent thresholds (|*ρ*| > 0.6) preferentially retain strong positive associations, the predominance of positive‐correlation edges should be interpreted as reflecting the most robust cooperative associations rather than the absence of negative interactions.

### 2.6 Differential Abundance, Enterotypes, and Functional Comparison

Differentially abundant features were identified by LEfSe (Segata et al. 2011), combining Kruskal–Wallis and pairwise Wilcoxon testing with linear discriminant analysis (LDA). For taxonomic profiles (phylum to species), MetaCyc pathways, and CAZy families, features with LDA > 2 and Benjamini–Hochberg FDR < 0.05 were retained.

Whether altitude corresponded to distinct enterotypes—statistically distinct community states defined by characteristic relative abundance profiles of co‐occurring genera (Arumugam et al. 2011)—was evaluated by fitting Dirichlet multinomial mixture (DMM) models to genus‐level abundance profiles (Holmes et al. 2012), with optimal cluster count selected via Laplace approximation. Robustness was cross‐checked with Partitioning Around Medoids (PAM) clustering. Altitude–enterotype association was assessed by Fisher's exact test, and discriminating genera were identified by Wilcoxon rank‐sum tests.

### 2.7 Fecal Metabolomics

Fecal aliquots were thawed on ice and homogenized. Metabolites were extracted with a methanol–water mixture (4:1, v/v) spiked with internal standards; after vortexing and ultrasonication, the suspensions were centrifuged (14,000×*g*, 20 min, 4°C) and the supernatants reserved for analysis. Pooled QC specimens, prepared from equal volumes of every study sample, were interspersed at regular intervals to monitor instrument drift.

Untargeted metabolomic profiling was conducted by Novogene Co. Ltd. (Beijing, China) on an UHPLC–MS/MS platform (Thermo Vanquish UHPLC; Waters ACQUITY BEH Amide HILIC column, 2.1 × 100 mm, 1.7 μm; Thermo Q Exactive HF‐X mass spectrometer; both positive and negative ESI; resolution 120,000 FWHM at m/z 200). Peak picking, alignment, and normalization were performed in Compound Discoverer 3.3, with putative identifications by spectral matching against HMDB, KEGG, and mzCloud (< 10 ppm mass accuracy; > 80% fragment similarity). Prior to multivariate modeling, feature intensities were log_2_‐transformed and Pareto‐scaled.

Orthogonal partial least squares‐discriminant analysis (OPLS‐DA) was implemented in the ropls R package (Thévenot et al. 2015), with model validity confirmed by 200 permutation tests (Wold et al. 2001; Trygg and Wold 2002). Metabolites were deemed significantly differential when satisfying a variable importance in projection (VIP) score—which quantifies each metabolite's weighted contribution to class separation in the OPLS‐DA model—greater than 1, Mann–Whitney FDR < 0.05, and—for volcano‐plot visualization—|log_2_FC| > 1. Pathway‐level enrichment of differential metabolites was assessed via hypergeometric testing against the KEGG‐annotated background detected in this dataset (FDR < 0.05).

We note that the standard untargeted UHPLC–MS/MS workflow employed here is not optimized for free SCFAs: their low molecular weights (60–102 Da), high polarity, suboptimal LC retention, and modest ESI ionization efficiency together render them largely undetectable by standard untargeted profiling. Reliable quantification requires specialized targeted methods (e.g., 3‐NPH derivatization‐based LC–MS or GC–MS), identified as a future direction in the Section 4.

### 2.8 MiMeNet‐Based Microbiome–Metabolome Integration

We employed MiMeNet, a multi‐task neural network for microbiome–metabolome integration (Reiman et al. 2021). Relative abundances of 17 differentially abundant species (selected from the 71 LEfSe hits by LDA > 3, prevalence ≥ 20% in at least one altitude group, and biologically interpretable functional annotation—primarily SCFA‐producing lineages and opportunistic pathogens of particular relevance to the altitude‐associated phenotype) served as input features to predict 15 differential metabolites (VIP > 1, |log_2_FC| > 1). Predictive accuracy was assessed by 10 rounds of 10‐fold cross‐validation (100 total runs), using Spearman *ρ* between measured and predicted abundances; metabolites with *ρ* > 0.3 were classified as well predicted. A 50‐iteration permutation null distribution was generated for benchmarking. Microbial feature importance was quantified by permutation‐based attribution scores, and Ward‐linkage bidirectional hierarchical clustering of the resulting attribution matrix was applied to delineate co‐functional microbe–metabolite modules. In parallel, pairwise Spearman correlations between key species and metabolites were calculated (retained at FDR < 0.05).

### 2.9 Isolation and Identification of Acid‐Producing Bacteria

Acid‐producing bacteria were recovered from high‐altitude fecal material on MRS agar containing 1% CaCO_3_ and incubated anaerobically at 37°C for 24–48 h. Colonies surrounded by clear calcium‐dissolution haloes exceeding 2 mm were purified through three rounds of streak plating and archived at −80°C in MRS broth with 50% glycerol. Each isolate was identified by Sanger sequencing of the 16S rRNA gene (27F/1492R) followed by BLAST against NCBI at a ≥ 97% identity cutoff. Phylogenetic relationships were reconstructed by neighbor‐joining in MEGA 11 (Kimura two‐parameter; 1000 bootstrap replicates).

### 2.10 Safety and Gastrointestinal Tolerance Screening

All isolates were tested for hemolytic activity on blood agar (37°C, 48 h, anaerobic) alongside *Staphylococcus aureus* CMCC (B) 26003 as a positive control; α‐ and β‐hemolytic strains were excluded. Antibiotic susceptibility was evaluated by Kirby–Bauer disk diffusion against six agents (ceftriaxone 30 μg, clarithromycin 15 μg, tetracycline 30 μg, chloramphenicol 30 μg, ampicillin 10 μg, ciprofloxacin 5 μg); after 18 h of incubation at 37°C, inhibition‐zone diameters were classified as susceptible, intermediate, or resistant per CLSI breakpoints (Humphries et al. 2021), and only strains showing no resistant phenotype across all six agents were advanced to the next screening tier. Acid tolerance (pH 3.0, 2.5, 2.0) and osmotic stress (0.3%, 0.6%, 1.0% NaCl) were assayed in MRS broth; growth at OD_600_ ≥ 0.3 within 24–48 h was considered positive. Antagonistic activity was determined by agar well diffusion against *Escherichia coli* CMCC (B) 44102, *Pseudomonas aeruginosa* CMCC (B) 10104, *S. aureus* CMCC (B) 26003, and *Salmonella enterica* serovar Paratyphi B CMCC (B) 50094 at 10^8^ CFU mL^−1^ on LB agar; 50 μL of cell‐free culture supernatant was dispensed into 6‐mm wells, with sterile MRS broth as a negative control. Zones of inhibition ≥ 10 mm after 18 h at 37°C were recorded as evidence of antimicrobial activity.

## 3 Results

### 3.1 Metagenomic Data Quality Control

Before downstream analysis, we first validated the metagenomic data quality. Metagenomic sequencing of 75 fecal samples generated 1376.9 Gb of raw data (9.18 billion reads). After quality filtering with fastp, 1366.4 Gb of high‐quality reads were retained (99.24% ± 0.41% retention rate), with each sample yielding 121 ± 16 million clean reads on average (Q20 > 98.5%, Q30 > 95.5%). Detailed quality metrics are provided in Table S1.

### 3.2 Altitude‐Associated Structural Divergence of the Forest Musk Deer Gut Microbiome

**FIGURE 1:**
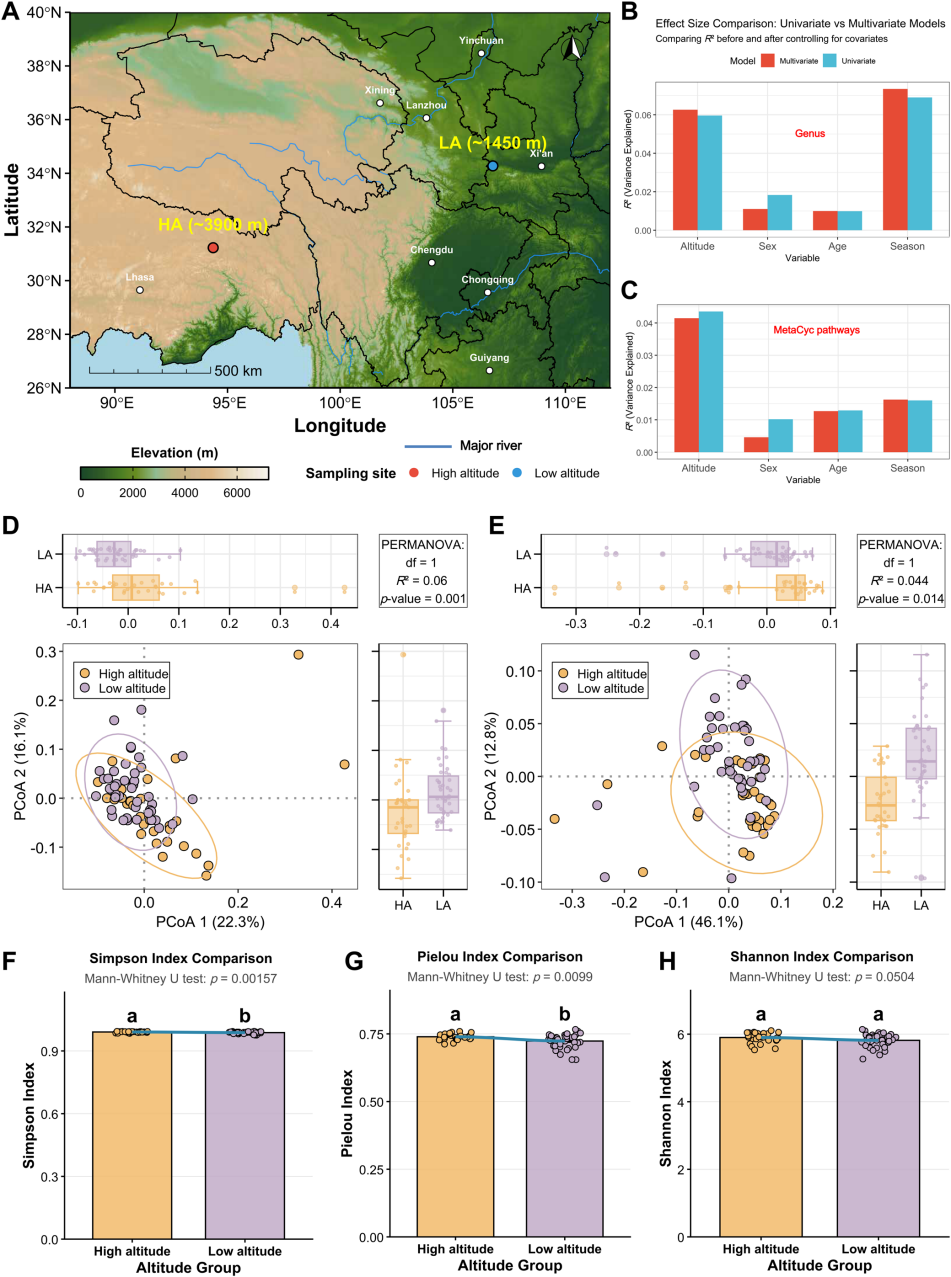
Altitude‐associated structural divergence of the captive forest musk deer gut microbiome. (A) Geographic distribution of sampling sites. Red circle: High‐altitude site (HA, ~3900 m, Tibet); blue circle: Low‐altitude site (LA, ~1450 m, Qinling Mountains, Shaanxi). Base map from standard map no. GS(2019)1822. (B, C) PERMANOVA effect size decomposition (*R* ^2^) for altitude, sex, age, and season on (B) genus‐level taxonomic and (C) MetaCyc functional pathway composition. Red bars: Multivariate models; blue bars: Univariate models. (D, E) PCoA based on Bray–Curtis dissimilarity for (D) genus‐level taxonomic composition (PCoA1: 22.3%) and (E) MetaCyc pathways (PCoA1: 46.1%). Orange: High altitude; purple: Low altitude; ellipses: 95% confidence intervals. (F–H) Alpha diversity comparison between groups: (F) Simpson, (G) Pielou evenness, (H) Shannon. Bars show means ± SEM; circles: Individual values; curves: Kernel density estimates. Mann–Whitney *U* test; letter annotations (a, b) denote significant differences (*p* < 0.05).

To assess the effects of altitude on the forest musk deer gut microbiome, we analyzed fecal samples from the Tibet Autonomous Region (high altitude, HA; 3900 m; *n* = 33) and Shaanxi Province (low altitude, LA; 1450 m; *n* = 42) (Figure 1A).

PERMANOVA based on Bray–Curtis dissimilarity revealed that altitude significantly explained variation in microbial community structure. At the genus level, both altitude and season significantly predicted community composition, explaining comparable fractions of variation (altitude *R* ^2^ = 0.060, *p* = 0.001; season *R* ^2^ = 0.069, *p* = 0.001), while sex and age did not reach significance (Figure 1B). PCoA revealed separation between altitude groups along PCoA1 (22.3%) (Figure 1D). At the MetaCyc functional pathway level, altitude was also significant (*R* ^2^ = 0.044, *p* = 0.014; PCoA1: 46.1%) (Figure 1C,E).

Alpha diversity analysis showed that the Shannon index did not differ significantly (HA: 5.900 ± 0.026; LA: 5.817 ± 0.031; *p* = 0.0504), but indices reflecting community evenness were significantly higher at high altitude: Simpson (0.991 ± 0.001 vs. 0.988 ± 0.001; *p* = 0.00157, Cohen's *d* = 0.51) and Pielou evenness (0.739 ± 0.002 vs. 0.724 ± 0.004; *p* = 0.0099, Cohen's *d* = 0.28) (Figure 1F–H). The high‐altitude gut microbiome thus did not differ in overall species diversity but exhibited a more even species abundance distribution.

### 3.3 Community Assembly Mechanisms Differ Between Altitude Groups

To partition the relative contributions of deterministic selection and stochastic processes to community assembly, we applied two complementary ecological null models, the Sloan neutral community model (NCM) and the normalized stochasticity ratio (NST), with the modified stochasticity ratio (MST) as an additional metric.

**FIGURE 2:**
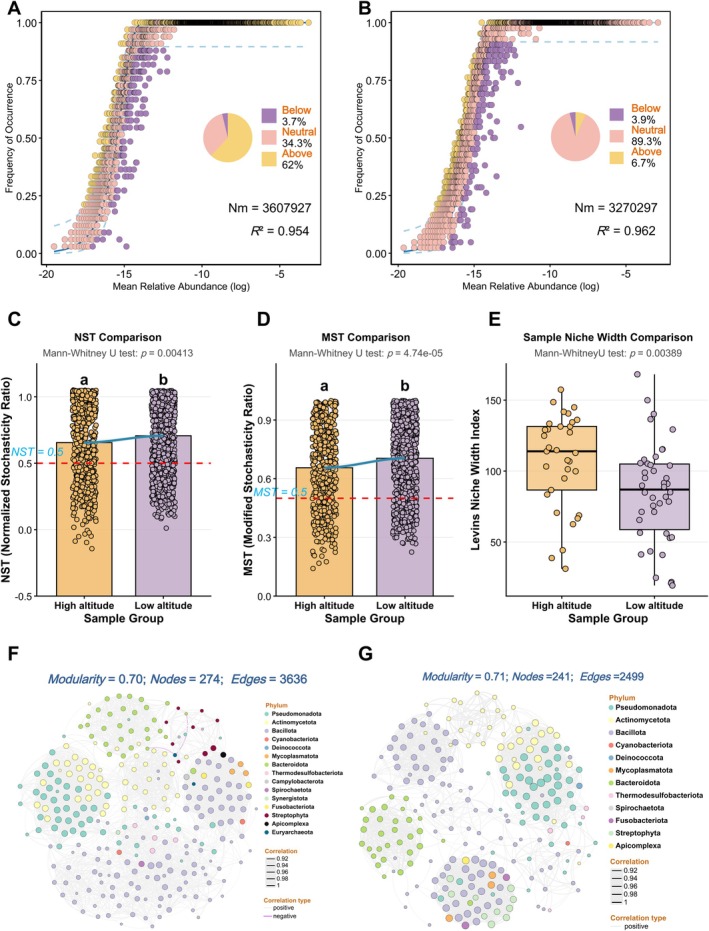
High‐altitude environment is associated with enhanced deterministic assembly and divergent co‐occurrence network topology. (A, B) Sloan neutral community model (NCM) fitting at genus level. Scatter plots show mean relative abundance (log‐transformed) versus occurrence frequency. Dashed lines: 95% neutral prediction intervals. Yellow: Taxa above predictions (positive selection); orange: Neutral; purple: Below predictions (negative selection). Pie charts: Category proportions. (A) High altitude; (B) Low altitude. (C, D) Stochasticity quantification: (C) normalized stochasticity ratio (NST, Ruzicka dissimilarity) and (D) modified stochasticity ratio (MST). Red dashed line: 50% threshold (above: Stochastic dominance). Mann–Whitney *U* test. (E) Levins' niche breadth comparison. (F, G) Species‐level co‐occurrence networks (|Spearman *ρ*| > 0.6, FDR < 0.01). Node colors: Phylum; node sizes proportional to mean relative abundance. Gray edges: Positive correlations; pink edges: Negative correlations. (F) High altitude; (G) Low altitude.

Neutral community model (NCM) analysis showed that both groups exhibited significant fit to the neutral model (LA: *R* ^2^ = 0.962; HA: *R* ^2^ = 0.954), but species distribution patterns differed markedly (Figure 2A,B). In the low‐altitude community, 89.3% of species fell within the 95% prediction interval of the neutral model, consistent with assembly dominated by stochastic dispersal and drift. In contrast, only 34.3% of species in the high‐altitude community conformed to neutral expectations, while 62.0% lay above the prediction interval—indicative of positive selection by environmental filtering—and the remaining 3.7% lay below.

NST analysis further quantified the relative contributions of assembly processes. The low‐altitude group NST (0.707 ± 0.268) was significantly higher than the high‐altitude group (0.655 ± 0.296) (Mann–Whitney *U* test: *p* = 4.13 × 10^−3^; Cohen's *d* = 0.19) (Figure 2C,D). Although NST values in both groups exceeded the 50% threshold—indicating that stochastic processes contributed substantially to assembly in both environments—the significantly lower NST in the high‐altitude group indicates a greater relative contribution of deterministic processes at high altitude (noting that NST cannot distinguish altitude per se from other site‐co‐varying environmental factors). The modified stochasticity ratio (MST) corroborated this pattern, with MST values significantly lower in the high‐altitude group (0.656 ± 0.211) than in the low‐altitude group (0.705 ± 0.196) (Mann–Whitney *U* test: *p* = 4.74 × 10^−5^; Cohen's *d* = 0.24). Consistently, Levins' niche breadth was significantly greater in the high‐altitude group (*p* = 0.00389; Figure 2E), indicating broader resource utilization.

Microbial co‐occurrence networks (|Spearman *ρ*| > 0.6, FDR < 0.01) revealed divergent topological structures between groups (Figure 2F,G). The high‐altitude network (274 nodes, 3636 edges) exhibited higher connection density than the low‐altitude network (241 nodes, 2499 edges): greater average degree (26.54 vs. 20.74), higher network density (0.097 vs. 0.086), shorter average path length (2.92 vs. 3.83), and higher clustering coefficient (0.808 vs. 0.786). Robustness analysis showed that the high‐altitude network maintained higher connectivity under both random (0.488 vs. 0.483) and targeted node removal (0.483 vs. 0.473), with a smaller robustness gap between attack modes (Δ = 0.005 vs. Δ = 0.010), suggesting lower dependence on hub nodes.

### 3.4 High‐Altitude Microbiomes Are Enriched in SCFA‐Producing Bacteria With Depletion of Opportunistic Pathogens

Having established structural divergence between altitude groups, we next identified the specific taxa driving this difference using linear discriminant analysis effect size (LEfSe), where LDA quantifies the effect size of each feature's contribution to group separation.

Across the 75 samples, Kraken2 classified an average of 20.4% of host‐filtered read pairs (range: 17.6%–34.5%) to at least one taxonomic rank; the substantial unclassified fraction (mean 79.6%) is consistent with published surveys of non‐model musk deer and other wild ruminant microbiomes and reflects the limited representation of musk deer‐associated taxa in current reference databases (Table S1).

**FIGURE 3:**
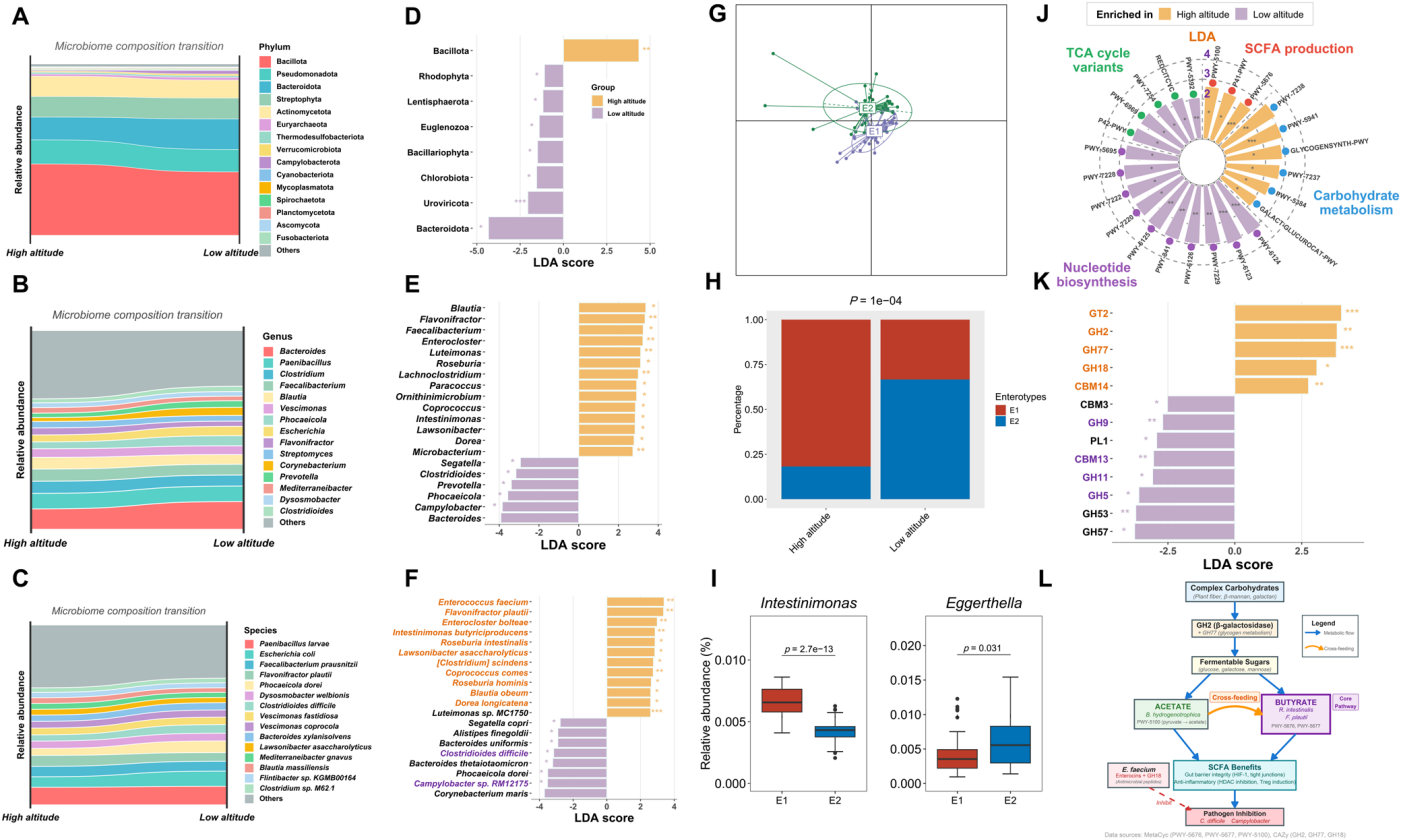
High‐altitude microbiome is enriched in SCFA‐producing bacteria with concurrent pathogen depletion and SCFA synthesis pathway enrichment. (A–C) Stacked composition profiles (top 15 of the 50 most abundant taxa per panel) at (A) phylum, (B) genus, and (C) species level. (D–F) LEfSe differentially abundant taxa (LDA > 2.0) at (D) phylum, (E) genus, and (F) species level. Orange: High‐altitude enriched; purple: Low‐altitude enriched. *FDR < 0.05; **FDR < 0.01; ***FDR < 0.001. Enterotype analysis (DMM modeling) is shown in panels G–I: (G) PCoA scatter plot showing E1/E2 separation (PAM clustering); (H) Enterotype distribution by altitude group (Fisher's exact test, *p* = 1 × 10^−4^); (I) Relative abundance of key driver genera (*Intestinimonas* and *Eggerthella*) between enterotypes. (J) Circular plot of differentially abundant MetaCyc pathways (LDA > 2.0, FDR < 0.05). Sector length proportional to LDA score; orange: High altitude; purple: Low altitude. Pathways grouped by functional category. (K) Differential CAZy enzyme families. CBM, carbohydrate‐binding module; GH, glycoside hydrolase; GT, glycosyltransferase; PL, polysaccharide lyase. (L) Conceptual model of the “complex carbohydrate degradation–SCFA synthesis–pathogen inhibition” functional cascade.

At the phylum level, the forest musk deer gut microbiome was dominated by *Bacillota*, *Bacteroidota*, *Pseudomonadota*, and *Actinomycetota* (cumulative ~80%). LEfSe analysis revealed *Bacillota* enrichment at high altitude (41.64% vs. 36.99%; LDA = 4.36, FDR = 2.78 × 10^−3^), while *Bacteroidota* was enriched at low altitude (17.82% LA vs. 13.27% HA; LDA = 4.35, FDR = 1.06 × 10^−2^); *Campylobacterota* showed a roughly 4‐fold reduction at high altitude (0.52% vs. 2.01%) (Figure 3A).

Genus‐ and species‐level analyses revealed systematic enrichment of SCFA‐producing bacteria at high altitude (Figure 3B,C,E,F). At the genus level, *Blautia* (5.88% vs. 4.87%), *Flavonifractor* (3.54% vs. 2.61%), *Faecalibacterium* (6.13% vs. 5.42%), *Roseburia*, *Coprococcus*, and *Intestinimonas* were all significantly enriched at high altitude (all LDA > 2.8, FDR < 0.05). Species‐level LEfSe analysis identified 71 differentially abundant species (LDA > 2, FDR < 0.05), of which 38 were enriched at high altitude (full list in Table S2). Confirmed SCFA producers enriched at high altitude included *Enterococcus faecium* (LDA = 3.37, FDR = 2.16 × 10^−3^), *Flavonifractor plautii* (LDA = 3.33, FDR = 2.16 × 10^−3^), *Roseburia intestinalis* , *Intestinimonas butyriciproducens*, *Coprococcus comes* , and *Blautia hydrogenotrophica* (Figure 3D–F; Figure S1A–G). In contrast, the low‐altitude group was enriched in multiple opportunistic pathogens, including *Campylobacter* sp. RM12175 (LDA = 3.53, FDR = 1.61 × 10^−2^) and *Clostridioides difficile* (LDA = 3.18, FDR = 1.50 × 10^−2^) (Figure 3F).

DMM modeling identified two optimal enterotypes (E1 and E2) at the genus level. Enterotype distribution was significantly associated with altitude (Fisher's exact test, *p* = 1 × 10^−4^): E1 predominated at high altitude, while E2 dominated at low altitude (Figure 3G,H). The high‐altitude‐associated E1 was characterized by elevated abundance of *Intestinimonas* (a confirmed butyrate producer), whereas E2 was driven by *Eggerthella* (Figure 3I).

### 3.5 Functional Pathway Analysis Reveals Enrichment of SCFA Synthesis Networks at High Altitude

To complement the taxonomic differences with functional‐level inference, we next mapped reads to MetaCyc metabolic pathways and CAZy carbohydrate‐active enzyme families to ask which functional modules differ between altitude groups.

A total of 49 differentially abundant MetaCyc pathways were detected between groups (LDA > 2, FDR < 0.05; full list in Table S3), with the high‐altitude group showing significant enrichment in SCFA synthesis pathways (Figure 3J). Specifically, acetyl‐CoA fermentation to butanoate (PWY‐5676; LDA = 2.42, FDR = 3.57 × 10^−4^)—the principal butyrate‐producing route—and pyruvate fermentation to acetate (PWY‐5100; LDA = 2.66, FDR = 1.10 × 10^−2^) were significantly enriched at high altitude. The concurrent enrichment of glycogen synthesis (GLYCOGENSYNTH‐PWY) and degradation (PWY‐5941) pathways indicates enhanced energy storage and mobilization capacity in the high‐altitude gut microbiota. The PWY‐5676 pathway, which drove the SCFA‐synthesis enrichment signal, comprises eight core enzymatic steps mediated by thiolase (*atoB*/*thl*), 3‐hydroxybutyryl‐CoA dehydrogenase (*hbd*), crotonase (*crt*), butyryl‐CoA dehydrogenase (*bcd*) with its electron‐transfer flavoprotein partners (*etfA*/*etfB*), and the terminal butyrate‐producing enzymes butyrate kinase (*buk*) or butyryl‐CoA: acetate CoA‐transferase (*but*). All eight core gene families were detected across our metagenomic assemblies, and their cumulative abundance was significantly higher at high altitude, consistent with the pathway‐level LDA enrichment.

CAZy annotation revealed corresponding differences in carbohydrate‐degrading enzyme profiles (Figure 3K). GT2 glycosyltransferase (LDA = 4.01, FDR = 3.04 × 10^−4^), GH2 β‐galactosidase (LDA = 3.85, FDR = 8.46 × 10^−3^), GH77 4‐α‐glucanotransferase (LDA = 3.79, FDR = 7.17 × 10^−5^), and GH18 chitinase (LDA = 3.10, FDR = 4.82 × 10^−2^) were significantly elevated at high altitude. GH2 and GH77 degrade complex carbohydrates to fermentable monosaccharides, supporting a functional cascade linking substrate liberation to the enriched SCFA synthesis pathways. The enrichment of an *E. faecium* ‐specific peptidoglycan synthesis pathway (PWY‐6471) was functionally consistent with its species‐level enrichment. The integrated functional cascade linking complex carbohydrate degradation to SCFA synthesis and downstream pathogen inhibition is summarized in Figure 3L.

### 3.6 SCFA‐Producing Bacteria Form Cooperative Networks and Show Negative Correlations With Pathogens

To examine how the altitude‐discriminating taxa interact with each other and with the depleted pathogens, we computed pairwise Spearman correlations across the 17 differentially abundant species identified above.

Spearman correlation analysis revealed highly structured patterns among differentially abundant species (Figure S1H). SCFA‐producing bacteria formed strong positive correlation networks: *I. butyriciproducens* and *Lawsonibacter asaccharolyticus* showed the highest correlation coefficient (*ρ* = 0.909, FDR = 1.78 × 10^−29^), followed by *L. asaccharolyticus*– *F. plautii* (*ρ* = 0.851) and *I. butyriciproducens*– *F. plautii* (*ρ* = 0.805). Within the genus *Roseburia*, *R. hominis* and *R. intestinalis* also exhibited strong positive correlation (*ρ* = 0.784), and together with *Blautia obeum* formed a functional module.

SCFA‐producing bacteria showed systematic negative correlations with *Campylobacter* pathogens. *E. faecium* was negatively correlated with *Campylobacter* sp. RM12175 (*ρ* = −0.411, FDR = 2.48 × 10^−4^), *Campylobacter* sp. RM8964 (*ρ* = −0.373), and *Campylobacter lanienae* (*ρ* = −0.327). *F. plautii* and *B. hydrogenotrophica* also showed negative correlations with multiple *Campylobacter* species (*ρ* range: −0.37 to −0.26).

### 3.7 Altitude‐Associated Fecal Metabolome Parallels Microbiome Functional Predictions

Having characterized the compositional divergence and functional‐pathway enrichment that distinguish high‐ from low‐altitude microbiomes, we next asked whether these sequencing‐based observations are paralleled at the metabolite level by profiling the fecal metabolome of all 75 individuals by untargeted UHPLC–MS/MS.

**FIGURE 4:**
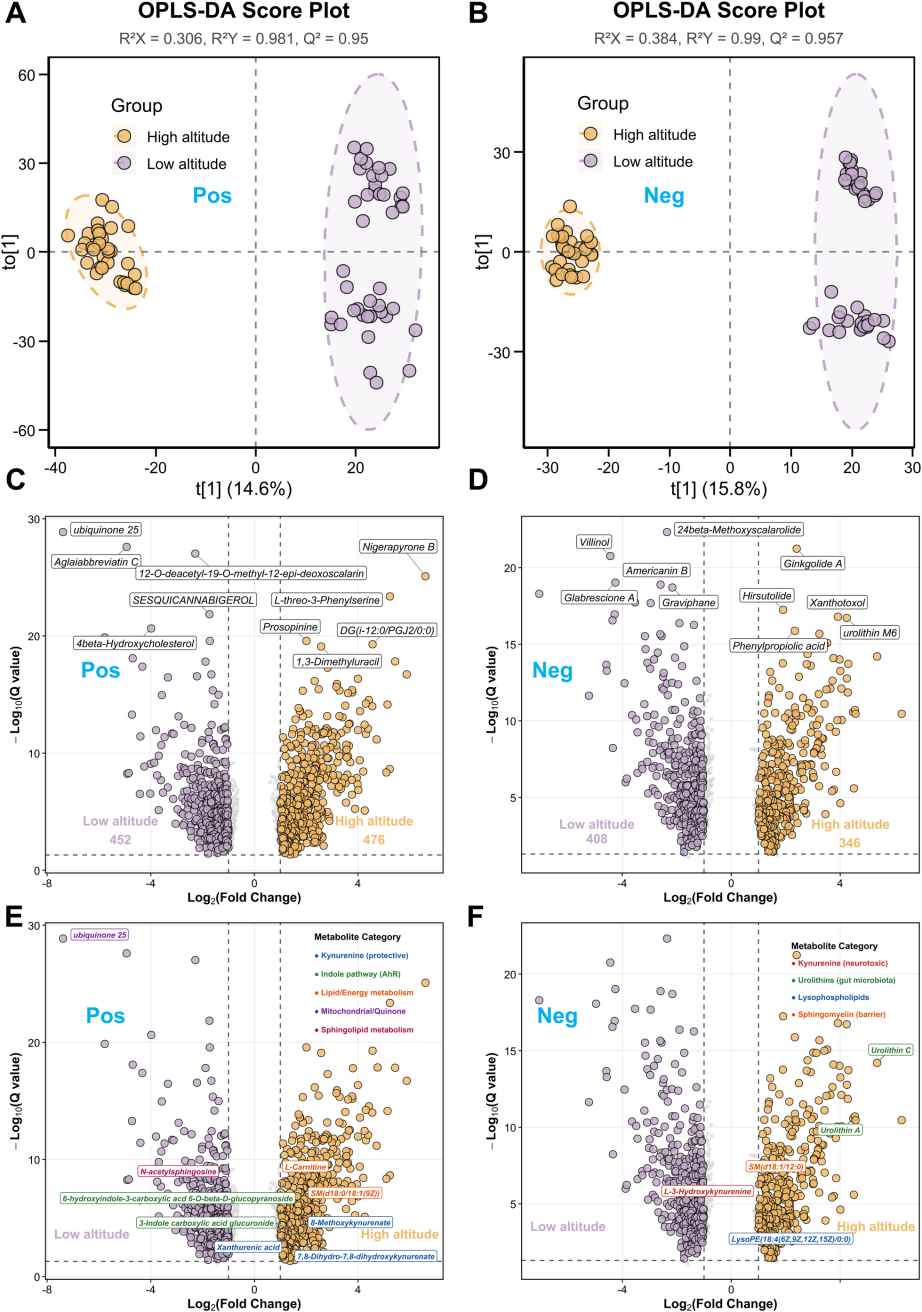
Altitude is associated with systematic differentiation of the fecal metabolome. (A, B) OPLS‐DA showing separation of fecal metabolic profiles between altitude groups. Horizontal axis: Predictive component t[1]; vertical axis: Orthogonal component to[1]. Model parameters (*R* ^2^ *X*, *R* ^2^ *Y*, *Q* ^2^) indicated; validity confirmed by 200 permutation tests. (A) Positive mode (*Q* ^2^ = 0.950); (B) Negative mode (*Q* ^2^ = 0.957). (C–F) Volcano plots of differential metabolites. Horizontal axis: Log_2_(Fold Change); vertical axis: −log_10_(FDR). Orange: Significantly upregulated at high altitude (log_2_FC > 1, FDR < 0.05); purple: Significantly upregulated at low altitude; gray: Non‐significant. (C, D) Positive and negative modes, respectively, with the most significant metabolites annotated by name. (E, F) The same positive and negative volcano plots with differential metabolites color‐coded by functional category. Chemical superclass composition and the top differential metabolites by VIP score are shown in Figure S3; KEGG pathway enrichment and the within‐pathway localization of upregulated metabolites are shown in Figure S4.

Untargeted metabolomics detected 6751 metabolites across both ionization modes (full list in Table S4). OPLS‐DA revealed robust separation between altitude groups (positive mode: *Q* ^2^ = 0.950; negative mode: *Q* ^2^ = 0.957; both *p* = 0.005) (Figure 4A,B). These *Q* ^2^ values fall within the range commonly reported for large‐cohort untargeted UHPLC–MS/MS studies of biofluids and feces with strong between‐group separation, where *Q* ^2^ values approaching 1 are considered indicative of robust predictive performance (Worley and Powers 2013), and a *Q* ^2^ above 0.5 is conventionally regarded as indicative of good predictive ability (Triba et al. 2015); model validity was further confirmed by 200 response‐permutation tests in which no permuted model exceeded the original. Applying three jointly required criteria—VIP > 1, FDR < 0.05, and |log_2_FC| > 1—we identified 1682 significantly differential metabolites between altitude groups (Figure 4C,D), of which 822 were upregulated at high altitude and 860 at low altitude. Differential metabolites grouped by functional category are highlighted in Figure 4E,F, while their chemical superclass composition and the top differential metabolites by VIP score are provided in Figure S3.

Differential metabolites revealed pronounced energy metabolism remodeling. Ubiquinone 25, a mitochondrial electron transport chain component, showed the most pronounced downregulation among all metabolites (log_2_FC = −7.38, VIP = 4.36, FDR = 1.37 × 10^−29^), while L‐carnitine, a key carrier for fatty acid β‐oxidation, was significantly upregulated (log_2_FC = 1.39, FDR = 5.42 × 10^−9^), together consistent with a shift toward fatty acid oxidation.

Lipid metabolites accounted for 30.9% of differential metabolites (520/1682). Because the underlying chemical‐class composition of the detected metabolome could itself be lipid‐biased, we tested whether lipids were disproportionately represented among differential features relative to their share of the full detected metabolome. Of the 6751 detected metabolites, 1847 (27.4%) were lipids (HMDB super‐class “Lipids and lipid‐like molecules”); the proportion among differential metabolites (520/1682 = 30.9%) was significantly higher than expected by chance (*χ* ^2^ = 14.26, df = 1, *p* = 1.59 × 10^−4^; odds ratio = 1.26), confirming a modest but statistically robust over‐representation of lipids among altitude‐discriminating features rather than a baseline‐composition artifact. Sphingomyelin metabolites were broadly upregulated at high altitude, including SM(d18:0/18:1(9Z)) (log_2_FC = 2.01), SM(d18:1/12:0) (log_2_FC = 1.10), and glucosylceramide (log_2_FC = 1.17), while *N*‐acetylsphingosine was significantly downregulated (log_2_FC = −2.36). The cholesterol metabolism intermediate 4β‐hydroxycholesterol was also markedly reduced (log_2_FC = −3.98).

The tryptophan–kynurenine pathway exhibited significant branch‐specific regulation. Products of the kynurenine aminotransferase (KAT) branch—the neuroprotective metabolites xanthurenic acid (log_2_FC = 1.12) and 8‐methoxykynurenate (log_2_FC = 1.77)—were upregulated at high altitude, whereas the neurotoxic metabolite L‐3‐hydroxykynurenine from the kynurenine monooxygenase (KMO) branch was significantly downregulated (log_2_FC = −1.41, FDR = 1.05 × 10^−5^).

Gut microbiota‐derived metabolites were significantly enriched at high altitude. Indole‐class aryl hydrocarbon receptor (AhR) ligands were upregulated, including 6‐hydroxyindole‐3‐carboxylic acid 6‐O‐beta‐D‐glucopyranoside (log_2_FC = 1.22) and 3‐indole carboxylic acid glucuronide (log_2_FC = 1.47). Urolithins—metabolites produced by gut microbiota transformation of ellagic acid—showed global upregulation: urolithin C (log_2_FC = 5.36, FDR = 6.27 × 10^−15^), urolithin A (log_2_FC = 4.54), urolithin M6 (log_2_FC = 4.24), and urolithin B (log_2_FC = 3.77).

KEGG pathway enrichment analysis identified four significantly enriched pathways (FDR < 0.05; Figure S4A): sphingolipid metabolism, arachidonic acid metabolism, tryptophan metabolism, and steroid hormone biosynthesis, with all 16 enriched metabolites upregulated at high altitude. Within arachidonic acid metabolism, enrichment spanned the cyclooxygenase (8‐iso‐PGF_2_α, log_2_FC = 1.67), cytochrome P450 (8,9‐DiHETrE, log_2_FC = 1.50), and 5‐lipoxygenase (leukotriene F4, log_2_FC = 1.38) branches. Steroid hormone upregulation included estrone sulfate (log_2_FC = 1.41) and pregnenolone sulfate (log_2_FC = 1.36).

### 3.8 Machine Learning Reveals Specific Associations Between SCFA‐Producing Bacteria and Altitude‐Enriched Metabolites

To investigate functional associations between gut microbiota and metabolites, Spearman correlation analysis was performed between 17 differentially abundant microbial species and 15 key metabolites, and MiMeNet neural network modeling was employed to validate the predictive capacity of the microbiome for the metabolome.

**FIGURE 5:**
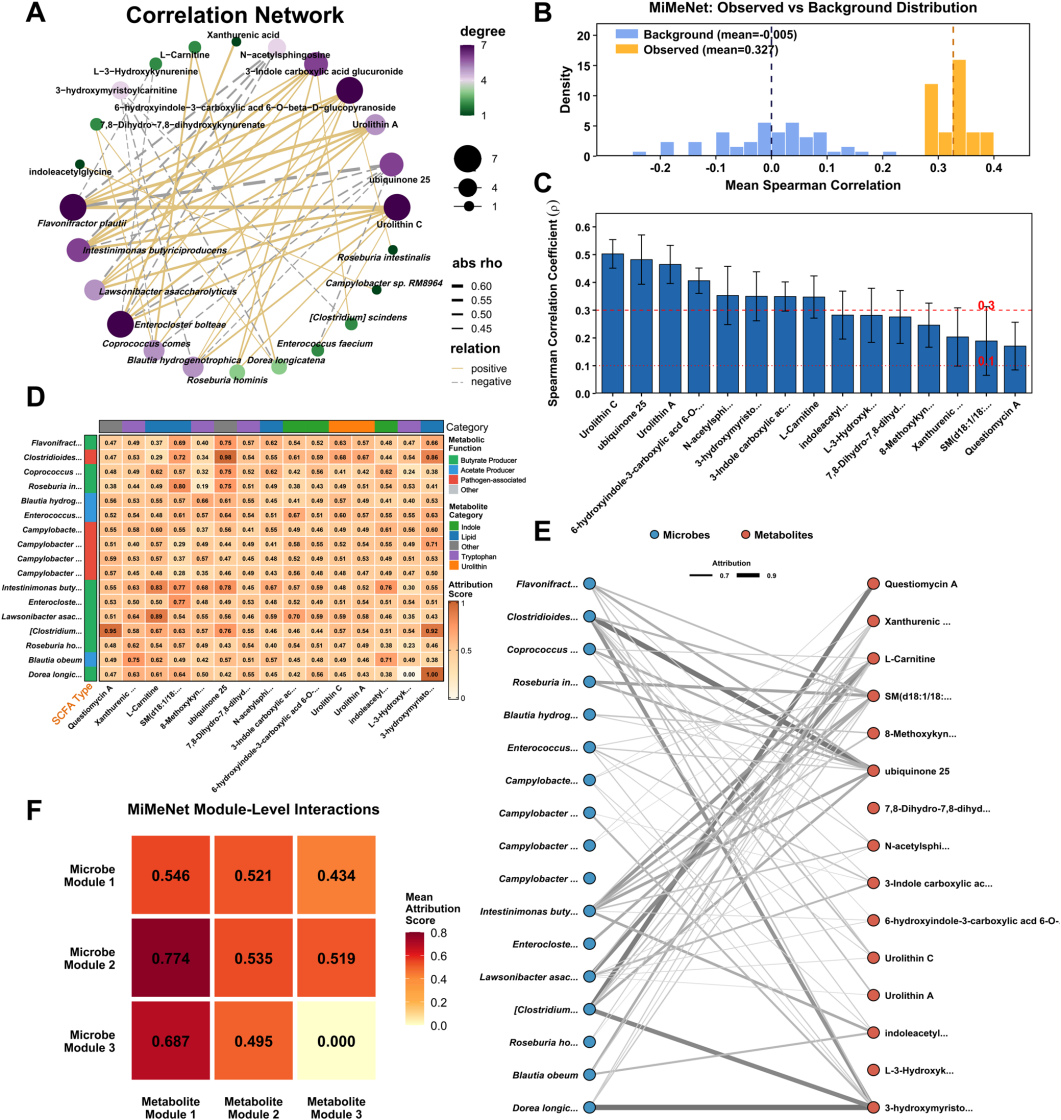
Microbiome–metabolome integration analysis. (A) Spearman rank correlation network between differentially abundant species and key metabolites (*p* < 0.05). Orange edges: Positive correlations; gray dashed edges: Negative correlations. (B) MiMeNet model validation: Density plot comparing observed (orange) and permuted background (blue) Spearman *ρ* distributions. (C) Cross‐validated predictive performance (mean Spearman *ρ*) for each metabolite. (D) Feature attribution score heatmap (scores 0–1). (E) Sankey diagram of microbe–metabolite attribution relationships (score > 0.7). (F) Module‐level interaction analysis: Mean attribution scores between microbial and metabolite modules.

A total of 184 significant associations (FDR < 0.05) were identified, comprising 107 positive and 77 negative correlations (Figure 5A). *Enterocloster bolteae* and *[Clostridium] scindens*—a key bile acid 7α‐dehydroxylating bacterium (Ridlon et al. 2006)—had the highest node degree (15), followed by *Roseburia hominis* and *Flavonifractor plautii* (14).

SCFA‐producing bacteria exhibited branch‐specific associations with the tryptophan–kynurenine pathway. The neurotoxic metabolite L‐3‐hydroxykynurenine showed significant negative correlations with nine SCFA‐producing bacteria, with *F. plautii* (*ρ* = −0.426) and *E. bolteae* (*ρ* = −0.420) showing the strongest associations. Conversely, the neuroprotective metabolite xanthurenic acid was positively correlated with 11 SCFA‐producing bacteria, led by *E. bolteae* (*ρ* = 0.512), *F. plautii* (*ρ* = 0.378), and *[Clostridium] scindens* (*ρ* = 0.363).

Urolithin metabolites formed the strongest positive correlation network with SCFA‐producing bacteria. *F. plautii* showed the highest correlation with urolithin C (*ρ* = 0.623, FDR = 2.14 × 10^−9^) and was also strongly correlated with urolithin A (*ρ* = 0.581). Other SCFA producers, including *I. butyriciproducens*, *L. asaccharolyticus*, and *E. bolteae*, showed similar positive correlations (*ρ* range: 0.497–0.546). In contrast, *Campylobacter* species were negatively correlated with urolithin metabolites (*ρ* range: −0.323 to −0.373). Ubiquinone 25 displayed an opposite association pattern, with *F. plautii* showing the strongest negative correlation (*ρ* = −0.597).

The overall MiMeNet predictive performance (mean *ρ* = 0.327) was significantly higher than the background distribution (mean *ρ* = −0.005), supporting genuine predictive associations (Figure 5B). Among the 15 differential metabolites, 8 (53.3%) achieved the good‐prediction criterion (*ρ* > 0.3). Urolithin metabolites showed the highest predictability: urolithin C (*ρ* = 0.503 ± 0.051) and urolithin A (*ρ* = 0.465 ± 0.069) ranked among the top three, together with ubiquinone 25 (*ρ* = 0.482 ± 0.089) (Figure 5C).

Feature attribution analysis identified key microbial drivers (Figure 5D). *Dorea longicatena* achieved the maximum attribution score (1.0) for 3‐hydroxymyristoylcarnitine, the strongest association among all 255 microbe–metabolite pairs. *[Clostridium] scindens* contributed highly to both questiomycin A (0.953) and 3‐hydroxymyristoylcarnitine (0.917). The strongest microbe–metabolite attribution relationships (score > 0.7) are visualized in Figure 5E, and bidirectional hierarchical clustering partitioned the 17 differential microbes and 15 differential metabolites into 3 functional modules each (Figure 5F).

### 3.9 Culturomics‐Based Strain Recovery Provides a Cultivable Resource With Broad‐Spectrum Antimicrobial Activity

Building on the compositional, functional, and metabolic patterns above, we next applied a culturomics‐based pipeline to recover a cultivable bacterial resource from this endangered host. The SCFA‐producing taxa most prominently enriched at high altitude in our metagenomic analysis—*Flavonifractor*, *Intestinimonas*, and *Roseburia*—remain technically challenging to recover under standard anaerobic cultivation; the cultivable strains obtained here therefore overlap with the altitude‐enriched guild at the functional rather than strictly taxonomic level. The choice to target acid‐producing bacteria was motivated by two converging lines of evidence: SCFA‐producing taxa constituted the most prominent compositional signature, and SCFA‐synthesis pathways the most prominent functional signature, of altitude‐associated divergence (Sections 3.4 and 3.5). Acid‐producing bacteria recoverable on CaCO_3_‐supplemented MRS agar therefore represented a functionally matched proxy for the altitude‐enriched guild.

**FIGURE 6:**
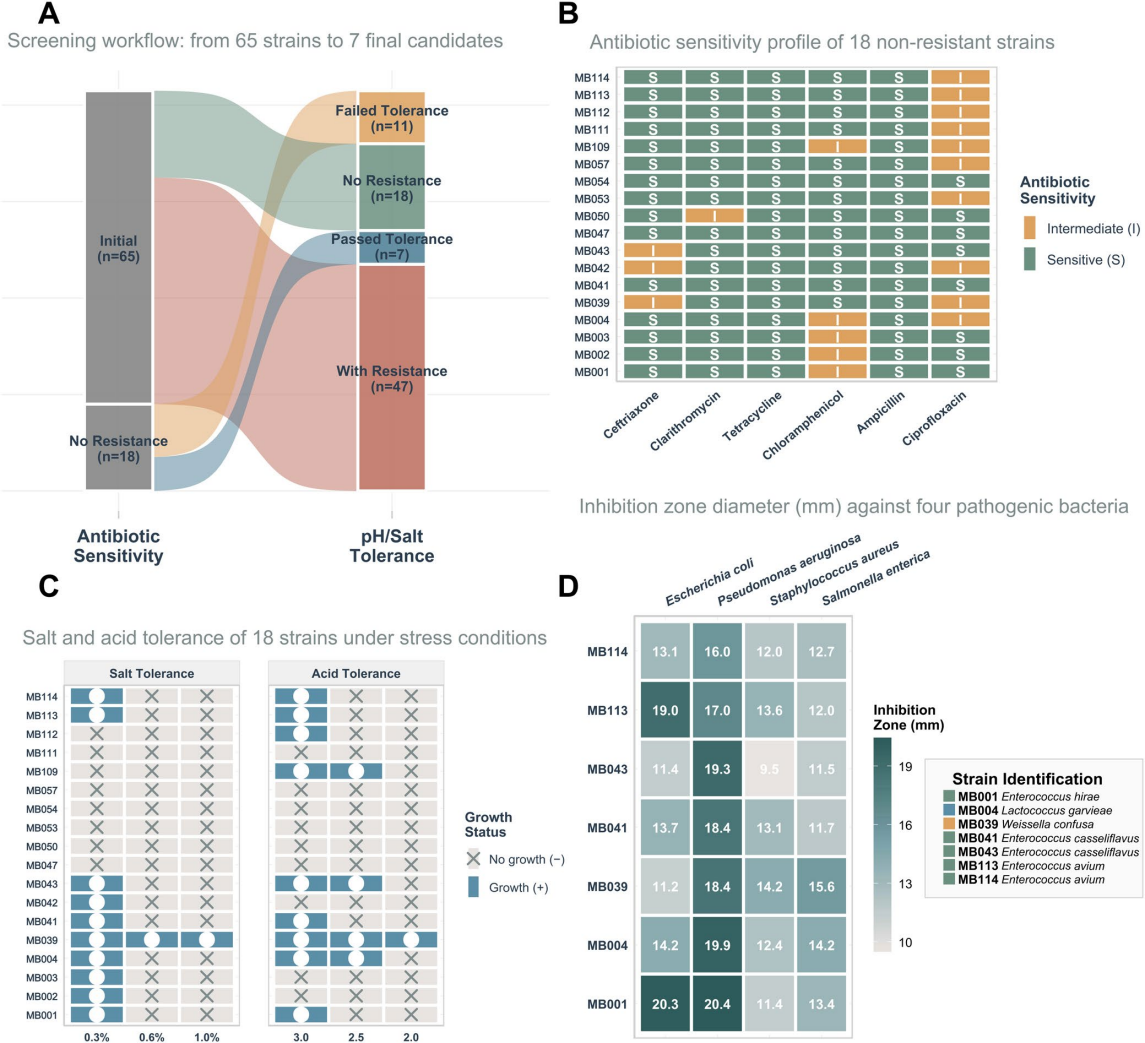
Multi‐tier safety and tolerance screening of cultivable strains from high‐altitude forest musk deer. (A) Sankey diagram of the multi‐tier screening workflow. From 112 acid‐producing isolates, 65 non‐hemolytic strains (γ‐hemolysis; 58.0%) underwent antibiotic susceptibility testing (18 retained, 27.7%), followed by gastrointestinal stress tolerance screening (7 final candidates, 10.8% overall passage rate). Taxonomic composition of isolated strains is shown. (B) Antibiotic susceptibility profiles of 18 non‐resistant strains against six antibiotics. Green: Susceptible (S); yellow: Intermediate (I). (C) Salt tolerance (0.3%–1.0% NaCl) and acid tolerance (pH 3.0–2.0) profiles. (D) Antimicrobial activity (inhibition zone diameter, mm) of seven final candidates against four indicator pathogens. *E. hirae* MB001 exhibited the strongest Gram‐negative activity ( *E. coli* : 20.3 mm; *P. aeruginosa* : 20.4 mm). The complete neighbor‐joining phylogenetic tree of 112 isolates and representative hemolysis phenotypes are shown in Figure S2.

A total of 112 acid‐producing strains were recovered from high‐altitude fecal samples and identified by 16S rRNA gene sequencing (Figure S2A). Phylogenetic analysis revealed nine species across three genera: *Enterococcus gallinarum* (*n* = 27, 24.1%) and *E. casseliflavus* (*n* = 26, 23.2%) dominated, followed by *E. hirae* (*n* = 20, 17.9%), *Weissella confusa* (*n* = 14, 12.5%), *E. avium* (*n* = 11, 9.8%), *E. faecium* (*n* = 10, 8.9%), *Lactococcus garvieae* (*n* = 2), *E. lactis* (*n* = 1), and *E. faecalis* (*n* = 1) (Figure S2B; Figure 6A). The taxonomic rank order of cultured isolates diverged from that in metagenomic data, in which *E. faecium* was the dominant *Enterococcus* species; this likely reflects the inherent selectivity of CaCO_3_‐based cultivation for the strongest acid producers.

Sequential screening evaluated key safety attributes and gastrointestinal stress tolerance (Figure 6A). Hemolytic activity testing on sheep blood agar revealed that 65 strains (58.0%) exhibited γ‐hemolysis (non‐hemolytic; Figure S2C), while 47 strains (42.0%) displayed α‐hemolysis (Figure S2D); no β‐hemolytic strains were detected. All α‐hemolytic strains were excluded. Of the 65 non‐hemolytic strains, antibiotic susceptibility testing retained 18 strains (27.7%) with no resistant phenotypes across all six tested antibiotic classes (Figure 6B). Gastrointestinal stress tolerance assays revealed pronounced inter‐strain variation: *W. confusa* MB039 maintained growth across all acid (pH 3.0–2.0) and salt (0.3%–1.0% NaCl) gradients, while other strains showed partial tolerance (Figure 6C). The overall passage rate from antibiotic susceptibility through antimicrobial activity screening was 10.8% (7 of 65 non‐hemolytic strains).

All seven final candidates— *E. hirae* MB001, *L. garvieae* MB004, *W. confusa* MB039, *E. casseliflavus* MB041, *E. casseliflavus* MB043, *E. avium* MB113, and *E. avium* MB114—exhibited broad‐spectrum antimicrobial activity against both Gram‐positive ( *S. aureus* ) and Gram‐negative ( *E. coli* , *P. aeruginosa* , *S. enterica* ) indicator pathogens (Figure 6D). *E. hirae* MB001 displayed the largest inhibition zones against Gram‐negative pathogens. Five of the seven candidates belonged to *Enterococcus*, the genus identified by metagenomic analysis as enriched at high altitude and negatively correlated with *Campylobacter* and *C. difficile* in co‐occurrence networks. Together, these isolates constitute a cultivable strain resource from this endangered host that provides a foundation for downstream mechanistic validation, controlled in vivo trials, and conservation‐oriented strain development that neither metagenomics nor metabolomics can supply alone.

## 4 Discussion

### 4.1 Patterns of Deterministic Community Assembly in the High‐Altitude Gut Microbiome

This study characterizes altitude‐associated divergence in the gut microbiome of captive forest musk deer, revealing compositional and functional patterns consistent with environmental filtering that parallel convergent microbiome configurations documented across phylogenetically diverse high‐altitude mammals (Tibetan humans: Ma et al. 2025; Zhao et al. 2024; yaks: Zhang et al. 2016; Tibetan pigs: Zeng et al. 2020; high‐altitude macaques: Li et al. 2023). Although NCM and NST analyses are well‐established frameworks in free‐living microbial ecology, our application to a captive endangered mammal system, in which animals are maintained under standardized husbandry, provides a particularly clean inferential window onto how strong environmental gradients reshape host‐associated community assembly. While research on golden snub‐nosed monkeys demonstrated deterministic‐dominated assembly under captivity (Zhang et al. 2025), our findings are consistent with altitude, together with co‐varying site‐specific factors, contributing substantially to this deterministic shift.

Two interpretive caveats warrant explicit acknowledgment. First, the modest individual *R* ^2^ values from PERMANOVA (altitude *R* ^2^ = 0.060; each predictor < 10%) are typical of gut microbiome surveys in wildlife; the biological significance of altitude rests not on total variance explained but on the consistency of its signal across independent analytical frameworks (PERMANOVA, NCM, NST/MST) and the downstream functional and metabolomic convergence documented across independent evidence layers. Second, the asymmetric distribution of taxa relative to the NCM prediction interval at high altitude—with most non‐neutral taxa positioned above rather than below—is consistent with environmental filtering acting primarily through selection *for* locally favored taxa rather than elimination of intolerant ones; we note that “positive selection” in the NCM sense refers specifically to taxa whose occurrence frequencies exceed the stochastic dispersal–drift expectation, and is distinct from the molecular‐evolution sense of positive selection (dN/dS > 1) on protein‐coding loci. Captive populations operate under effectively closed metacommunities, however, and alternative contributors to elevated occurrence frequencies (host‐specific recruitment, shared facility‐level inputs) cannot be fully excluded by the present design.

The two altitude‐associated enterotypes, with the high‐altitude E1 driven by the butyrate producer *Intestinimonas*, link altitude‐related community assembly to SCFA‐producing functional guilds. The remarkably strong positive co‐occurrence among *I. butyriciproducens*, *F. plautii* , and *L. asaccharolyticus* (*ρ* = 0.81–0.91) and the metabolic cross‐feeding cascade from complex carbohydrate degradation (GH2, GH77) through SCFA synthesis (PWY‐5676, PWY‐5100) point to a functionally integrated consortium rather than independently enriched taxa. Whether this reflects true host–microbiome co‐evolution or phenotypic plasticity of a flexible microbiome responding to altitude‐associated intestinal conditions remains a key open question—one that future longitudinal tracking of relocated individuals and reciprocal fecal microbiota transplantation (FMT) experiments can address.

### 4.2 Convergent Enrichment of SCFA‐Producing Bacterial Networks: A Community‐Wide Response to High‐Altitude Metabolic Demands

The convergent enrichment of butyrate‐producing taxa— *F. plautii* , *I. butyriciproducens*, *R. intestinalis* , *C. comes* , and *E. faecium* —in the high‐altitude microbiome is consistent with a community‐wide response across phylogenetically diverse high‐altitude mammals. Meta‐analysis of Qinghai–Tibet Plateau populations confirmed elevated butyrate‐producing bacteria, primarily from *Lachnospiraceae* and *Ruminococcaceae* (Zhao et al. 2024). That the same functional guild—rather than the same taxa—is repeatedly enriched across mammalian orders separated by > 80 million years of divergence suggests convergence at the metabolic pathway level, plausibly linked to altitude‐associated hypoxia. This pattern parallels convergent genomic signatures (e.g., in *EPAS1* and HIF pathway genes) across high‐altitude mammalian genomes (Wu et al. 2020), but extends this convergent pattern to host‐associated microbial communities.

Deep metagenomic sequencing identified co‐enriched components consistent with a metabolic cascade linking polysaccharide degradation (GH2 β‐galactosidase, GH77 4‐α‐glucanotransferase) to SCFA synthesis pathways (PWY‐5100 for acetate, PWY‐5676 for butyrate), further enhanced by metabolic cross‐feeding between primary degraders and butyrate producers (Belenguer et al. 2006). The independent enrichment of the *E. faecium* ‐specific peptidoglycan synthesis pathway (PWY‐6471) provides species‐resolved functional validation. This integrated co‐enrichment confirms a functionally coherent degradation‐to‐fermentation network rather than merely elevated abundance of individual producers; direct flux measurements are needed to confirm pathway connectivity.

Butyrate, if produced at correspondingly elevated levels, fulfills multiple functions relevant under high‐altitude conditions. As the preferred energy substrate for colonocytes, it maintains epithelial energy homeostasis under hypoxia through HIF signaling (Kelly et al. 2015), enhances intestinal barrier integrity by upregulating tight‐junction proteins (Qi et al. 2025), and exerts systemic anti‐inflammatory activity via NF‐κB and histone deacetylase (HDAC) inhibition (Martin‐Gallausiaux et al. 2021). In rodent models, butyrate attenuates hypoxia‐induced pulmonary vascular remodeling (Karoor et al. 2021). Beyond these metabolic functions, SCFA‐producing bacteria may also contribute to colonization resistance through pH‐mediated pathogen inhibition, as discussed in Section 4.4. The forest musk deer, the first endangered moschid examined in this context, extends this emerging cross‐species pattern of SCFA‐enriched microbiome configurations at high altitude. The identification of a metabolic cascade linking polysaccharide degradation to SCFA synthesis and downstream pathogen inhibition provides a mechanistic rationale for dietary fiber supplementation strategies and targeted probiotic formulations aimed at reconstituting this functional module in low‐altitude or dysbiotic captive herds.

### 4.3 Altitude‐Associated Metabolic Remodeling of the Tryptophan–Kynurenine Pathway

Our data reveal a systematic association between SCFA‐producing bacteria and branch‐specific modulation of the tryptophan–kynurenine pathway. Butyrate producers enriched at high altitude— *F. plautii* , *E. bolteae*, *B. hydrogenotrophica* , and *R. intestinalis* —showed significant negative correlations with the neurotoxic metabolite L‐3‐hydroxykynurenine and positive correlations with the neuroprotective metabolite xanthurenic acid. This concurrent enrichment of neuroprotective‐branch metabolites and depletion of neurotoxic‐branch metabolites suggests metabolic reprogramming associated with gut microbiome composition, with causality likely operating bidirectionally: host‐driven kynurenine shifts at high altitude could in turn favor SCFA‐producing taxa. The most likely mechanistic basis involves butyrate regulation of indoleamine 2,3‐dioxygenase 1 (*IDO1*), the rate‐limiting enzyme of the kynurenine pathway: butyrate inhibits *IDO1* expression through STAT1 phosphorylation inhibition and direct *IDO1* repression as an HDAC inhibitor (Martin‐Gallausiaux et al. 2018). Under high‐altitude hypoxia, where HIF‐1α‐dependent regulation of *IDO1* and tryptophan 2,3‐dioxygenase (TDO) modulates tryptophan catabolism (Huang et al. 2020), the interplay between host hypoxia signaling and microbiome‐derived butyrate may favor the KAT (neuroprotective) over the KMO (neurotoxic) branch. Additionally, butyrate can promote tryptophan shunting toward indole pathways, enhancing AhR signaling (Agus et al. 2018), consistent with the observed upregulation of indole‐class AhR ligands.

This pattern has direct implications for forest musk deer conservation. Captive musk deer exhibit high susceptibility to gastrointestinal diseases and stress‐related mortality (Jiang et al. 2021), and L‐3‐hydroxykynurenine is an established generator of reactive oxygen species inducing neuronal apoptosis and exacerbating inflammatory cascades (Schwarcz et al. 2012). The observed neuroprotective shift represents a microbiome‐mediated mechanism that may mitigate oxidative and neuroinflammatory stress in a species already challenged by captivity‐associated stressors. Notably, MiMeNet revealed differential predictive performance across metabolite classes: microbiota‐dependent metabolites such as urolithins were highly predictable, while kynurenine pathway metabolites were less so, reinforcing a two‐tier model in which kynurenine concentrations are primarily governed by host *IDO1*/TDO activity with the microbiome exerting indirect modulatory influence—most likely via butyrate‐mediated *IDO1* modulation. To our knowledge, this is the first report of gut microbiome–kynurenine pathway associations in a non‐model endangered species.

### 4.4 Altitude‐Associated Urolithin Enrichment and *Enterococcus*‐Mediated Colonization Resistance: From Sequence‐Inferred Networks to Culture‐Validated Isolates

The significant enrichment of urolithin metabolites—urolithin A, B, C, and M6—in the high‐altitude fecal metabolome warrants particular attention. Urolithins, produced by gut microbiota transformation of dietary ellagic acid, possess multiple physiological activities, including mitophagy activation, intestinal barrier enhancement, and anti‐inflammatory effects (D'Amico et al. 2021). Recent identification of the urolithin C dehydroxylase (*ucd*) operon in *Enterocloster* species (Pidgeon et al. 2025) is directly consistent with our results: MiMeNet identified urolithin C and A as the most predictable metabolites, and *E. bolteae* was among the microbes most closely associated with urolithins. Although *F. plautii* and *I. butyriciproducens* are not themselves urolithin producers, their SCFA‐mediated gut lumen acidification may create conditions favorable for *ucd*‐harboring *Enterocloster*. Urolithin A induces mitophagy via the PINK1–Parkin pathway (Ryu et al. 2016) and improves mitochondrial health in clinical trials (Singh et al. 2022); it also enhances barrier function through AhR–Nrf2 signaling (Singh et al. 2019), and the concurrent upregulation of indole‐class AhR ligands suggests multi‐pathway AhR activation under high‐altitude conditions. The concurrent urolithin enrichment, ubiquinone 25 downregulation, and L‐carnitine upregulation reflect an energy metabolism shift toward fatty acid oxidation under high‐altitude hypoxia.

The reciprocal distribution of beneficial bacteria and pathogens between altitude groups— *E. faecium* enriched at high altitude versus *C. difficile* and *Campylobacter* enriched at low altitude—suggests competitive exclusion or direct antimicrobial mechanisms. *E. faecium* harbors a diverse bacteriocin repertoire (enterocins A, B, P, L50A/B, E‐760) with broad‐spectrum activity against pathogens and efficacy against *Clostridioides*‐related gastrointestinal colitis (Franz et al. 2007; Todorov et al. 2020); the *E. faecium* P2 strain has recently been shown to inhibit *C. difficile* growth and biofilm formation in vitro and reduce intestinal damage in mouse models (Cui et al. 2025). Our culturomics data support this hypothesis: all seven final candidates exhibited broad‐spectrum antimicrobial activity, with comparable profiles across multiple *Enterococcus* species, suggesting functional redundancy buffering colonization resistance. The synergistic enrichment of SCFA‐producing bacteria and *E. faecium* at high altitude may reinforce colonization resistance through complementary mechanisms: butyrate enhances barrier integrity, lactic acid lowers pH to inhibit *C. difficile* spore germination (Pike and Theriot 2021), and bacteriocins directly kill susceptible pathogens. Notably, *T. pyogenes* —the predominant pathogen causing high mortality in captive forest musk deer—was detected at extremely low overall abundance across both altitude groups in our metagenomic data, consistent with its role as an opportunistic rather than commensal pathogen; whether SCFA‐mediated colonization resistance additionally suppresses *T. pyogenes* colonization warrants targeted investigation.

However, the dual nature of *Enterococcus* species warrants caution: epidemic strain succession of hospital‐acquired vancomycin‐resistant enterococci is mediated by bacteriocin acquisition (Kommineni et al. 2015). Notably, no *E. faecium* strains passed our four‐tier screening, reflecting elevated intrinsic resistance rates in this species. The four‐tier pipeline—hemolysis, antibiotic susceptibility, gastrointestinal stress tolerance, and pathogen antagonism—represents one of the most stringent safety screening protocols applied to an endangered species and provides a methodological template directly transferable to probiotic development programs for other conservation‐priority taxa. *W. confusa* MB039 (pan‐tolerant) and *E. hirae* MB001 (strong Gram‐negative activity) represent the most promising candidates. Whole‐genome sequencing of the final candidates—particularly *E. hirae* MB001 and *E. avium* MB113/MB114—is warranted for thorough safety characterization, biosynthetic gene cluster annotation, and exclusion of transferable resistance elements before in vivo trials.

### 4.5 Broader Conceptual Contributions

Although grounded in a specific endangered species system, our findings speak to several general questions in microbial ecology and conservation biology. First, the convergent enrichment of SCFA‐producing bacteria across phylogenetically diverse high‐altitude mammals separated by > 80 million years of divergence suggests that community‐level convergence operates primarily at the metabolic pathway level rather than at the species level—consistent with theoretical frameworks positing that functional redundancy permits different species to fulfill equivalent ecosystem roles (Louca et al. 2018; Groussin et al. 2017)—and extends this convergent pattern from host genomes to host‐associated microbial communities.

Second, our integration of NCM and NST analyses with downstream metabolomics and culturomics provides a template for quantitatively dissecting how strong environmental gradients reshape community assembly in host‐associated systems. The observation that only 34.3% of high‐altitude species fell within neutral prediction envelopes—compared with 89.3% at low altitude—constitutes, to our knowledge, the first quantitative demonstration that a natural altitudinal contrast can shift assembly from stochastic toward deterministic dominance within standardized captive management. Extending these frameworks, originally developed on free‐living microbial systems (Stegen et al. 2012; Ning et al. 2019), to host‐associated systems under controlled husbandry offers a cleaner inferential window on how environmental filtering interacts with community assembly dynamics.

Third, the parallel shifts in SCFA‐producing taxa, their cognate metabolic pathways, and downstream microbiome‐derived metabolites (urolithins, tryptophan‐derived neuroactive compounds) are consistent with the gut microbiome participating in—rather than merely responding to—host‐level physiological responses to environmental stress, relevant to hologenomic theory (Bordenstein and Theis 2015). The multi‐omics concordance documented here establishes a testable framework in which future reciprocal‐transplant or longitudinal studies can disentangle community‐level co‐response from reciprocal host–microbiome interactions. Methodologically, the integration employed here—combining deep shotgun metagenomics with neutral‐model‐based assembly analysis, untargeted metabolomics, and culturomics‐based strain recovery—offers a template for conservation metagenomics studies seeking to move beyond taxonomic cataloging toward mechanistic inference and actionable outputs, bridging a persistent gap between sequencing‐based surveys and the characterized strains required for downstream conservation applications.

### 4.6 Translating Ecological Insight Into Conservation Practice

The integrative approach demonstrated here is not restricted to forest musk deer. The altitude‐informed facility‐siting strategy is applicable to other captive breeding programs along elevational gradients—such as giant pandas ( *Ailuropoda melanoleuca* ) at facilities ranging from ~500 to ~2800 m and golden snub‐nosed monkeys, whose captive microbiomes vary with facility conditions (Zhang et al. 2025)—and the culturomics‐based screening pipeline is directly transferable to probiotic development for other captive ruminants (Hameed and Nazir 2025). We propose four management recommendations, stratified by evidence strength and the additional validation required for operational deployment.

#### 4.6.1 Altitude‐Informed Facility Siting (Reasonable Extrapolation)

The high‐altitude gut microbiome harbors a functionally coherent SCFA‐producing consortium with enhanced colonization resistance, substantially attenuated at low altitude where antibiotic resistance genes and infection‐associated pathways are enriched. When establishing new breeding facilities, managers should consider that higher‐altitude sites may passively promote the assembly of health‐protective microbiome configurations. Practical implementation should proceed in conjunction with validation across intermediate elevational gradients and matched‐altitude multi‐site confirmation (Guo et al. 2025).

#### 4.6.2 Species‐Specific FMT Protocol Design (Speculative Extension)

The differentiated profiles provide a rational basis for donor selection: healthy adults from high‐altitude populations with high SCFA‐producer abundance and low pathogen burden merit consideration as preferred donors, with culturomics‐characterized strains as quality‐control references. Operational deployment would require controlled donor–recipient matching trials.

#### 4.6.3 Host‐Derived Probiotic Development (Directly Supported in Vitro)

The combined metagenomic and culturomics validation of *Enterococcus* species, together with established probiotic precedent in livestock and endangered ruminants (Markowiak and Śliżewska 2018; Hameed and Nazir 2025), positions the seven validated candidates—particularly *W. confusa* MB039 and *E. hirae* MB001—as starting points for host‐specific probiotic formulations. In vivo efficacy and safety in forest musk deer await confirmation in controlled feeding trials.

#### 4.6.4 Non‐Invasive Health Monitoring (Reasonable Extrapolation)

Urolithin metabolites, fecal SCFA profiles, and kynurenine pathway metabolite ratios (e.g., xanthurenic acid/L‐3‐hydroxykynurenine) represent candidate biomarkers for gut health assessment, requiring longitudinal within‐individual validation to establish predictive utility.

These recommendations rest on several assumptions: that altitude‐associated microbiome patterns reflect environmental filtering rather than facility‐specific confounders; that in vitro tolerance and antagonism translate to in vivo benefits; that population‐level metabolite signatures retain individual‐level predictive value; and that microbiome benefits of higher altitudes outweigh the practical considerations of siting facilities at elevation. Users should treat these as informed starting points for operational trials, not as validated management prescriptions.

### 4.7 Limitations and Future Research Priorities

The study compares two geographically distinct facilities, so altitude is confounded with co‐varying variables, including latitude, climate, vegetation, water source, and potentially genetic background (Fan et al. 2019), as well as fine‐scale site‐specific variables (soil‐derived microbial exposure, feed batch variation, facility hardware, operational routines, water mineral composition). We have therefore framed our findings in terms of environmental filtering and altitude‐associated patterns rather than direct causal claims of altitude‐driven adaptation. Definitive causal dissection would require reciprocal transplant experiments, multi‐site sampling with matched altitudes, or within‐site sampling across continuous elevational gradients.

Beyond study‐design limitations, three additional layers of methodological indirectness warrant acknowledgment. First, our multi‐omics integration is correlational and cannot establish direct causal metabolic links between specific microbial species and their putative products. Second, functional inferences are based on genomic potential rather than validated expression or flux measurements; confirmation of in vivo activity would require metatranscriptomic, metaproteomic, or stable‐isotope probing validation. Third, our culturomics‐based screening was conducted by isolating acid‐producing bacteria on CaCO_3_‐supplemented media rather than by targeted recovery of the specific altitude‐enriched SCFA producers identified metagenomically; the bridge between the altitude‐enriched guild ( *F. plautii* , *I. butyriciproducens*) and the culturomics‐validated isolates (*Enterococcus* and *Weissella* species) is thematic rather than strictly causal.

A specific methodological limitation of the metabolomic profiling is that the untargeted UHPLC–MS/MS workflow does not reliably capture free SCFAs. A targeted search of all 6751 annotated features for the six canonical SCFAs—acetic, propionic, butyric, isobutyric, valeric, and isovaleric acids—confirmed that none was detected. Direct metabolite‐level validation of the metagenomically inferred SCFA‐synthesis enrichment therefore awaits a dedicated targeted SCFA assay (e.g., 3‐NPH derivatization‐based LC–MS or GC–MS). The multi‐omics SCFA narrative nonetheless remains internally consistent across three converging metagenomic granularities: the pathway level (PWY‐5676 significantly enriched at high altitude), the gene‐family level (all eight core butanoate‐synthesis genes at coordinated higher abundance at high altitude), and the taxonomic level (*Flavonifractor*, *Intestinimonas*, and *Roseburia* all enriched at high altitude).

The cross‐sectional design further precludes causal inference; longitudinal tracking would clarify whether differences represent plastic responses or stable adaptations. Although our sample size (*n* = 75) provided sufficient power for moderate‐to‐large effect sizes, it may have been underpowered for subtler differences. Future priorities include multi‐site studies encompassing intermediate elevations and larger sample sizes; wild–captive comparisons (Moeller et al. 2014) to delineate altitude versus captivity effects; dietary fiber manipulation and controlled FMT trials testing whether high‐altitude microbiomes confer protective phenotypes to low‐altitude recipients; host transcriptomics and immunological profiling—particularly *IDO1*/KAT/KMO expression—to elucidate molecular mechanisms; and establishment of a forest musk deer gut microbiome gene catalog and microbial biobank as foundational infrastructure for conservation metagenomics in endangered musk deer and other wild ruminants.

## 5 Conclusions

Altitude emerges here as an environmental gradient associated with distinct captive gut microbiome configurations, function, and colonization resistance patterns—with direct, practical implications for how endangered species are managed in captivity. In high‐altitude forest musk deer, the gut microbiome harbors a functionally coherent guild of SCFA‐producing bacteria centered on *F. plautii* , *I. butyriciproducens*, and *E. faecium* , whose assembly is consistent with deterministic processes and which exhibit antagonistic co‐occurrence with opportunistic pathogens. These compositional shifts are coupled with host metabolic remodeling, including urolithin biosynthesis and branch‐specific modulation of the tryptophan–kynurenine axis. This integrated microbiome–metabolome architecture constitutes a coupled altitude‐associated functional module that is substantially attenuated in low‐altitude conspecifics, where antibiotic resistance genes and infection‐associated pathways are relatively enriched. Although altitude co‐varies with other geographic factors in this design, the internal consistency across taxonomic, functional, metabolomic, and culture‐based evidence layers supports a biologically meaningful environmental association, while acknowledging that definitive causal attribution awaits future reciprocal transplant or within‐site gradient studies.

By coupling deep metagenomics with culturomics‐based strain recovery and a four‐tier phenotypic screening pipeline, we advance in silico network predictions toward safety‐validated cultivable strains with confirmed gastrointestinal tolerance and broad‐spectrum pathogen‐antagonistic activity in vitro—narrowing the “sequencing‐to‐strain” gap that limits the translational utility of most conservation metagenomics studies. The resulting strain library, keystone species, and metabolic biomarkers provide actionable targets for facility siting, FMT donor selection, probiotic supplementation, and non‐invasive health surveillance. These findings point to altitudinal gradients as a potentially important yet previously underexplored dimension of captive management for endangered species, and illustrate how characterizing altitude‐associated host–microbiome divergence can inform evidence‐based conservation: by delineating how environmental gradients shape functional microbial patterns, we gain the capacity to design interventions informed by ecological context, ultimately improving captive health outcomes and reintroduction success across endangered species worldwide.

## Funding

This work was supported by the Science and Technology Project of Sichuan Province (Grant No. 2025ZNSFSC0252).

## Ethics Statement

All sample collection strictly adhered to the principle of minimal disturbance. Fecal samples were collected only after target individuals had naturally defecated and moved away, ensuring zero interference with individual forest musk deer and full compliance with animal welfare ethical standards.

## Conflicts of Interest

The authors declare no conflicts of interest.

## Supporting information


**Figure S1:** Enrichment of SCFA‐producing bacteria and species correlation network. (A–G) Relative abundance of key SCFA‐producing bacteria between altitude groups: (A) *Enterococcus faecium* , (B) *Flavonifractor plautii* , (C) *Enterocloster bolteae*, (D) *Intestinimonas butyriciproducens*, (E) *Roseburia intestinalis* , (F) *Lawsonibacter asaccharolyticus*, (G) *[Clostridium] scindens*. Bars: means ± SEM; circles: individual values. Orange: high altitude; purple: low altitude. (H) Spearman correlation heatmap with hierarchical clustering among 17 differentially abundant species.

**Figure S2:** Phylogenetic identification and hemolytic activity screening of acid‐producing isolates. (A) Neighbor‐joining phylogenetic tree (16S rRNA) of 112 acid‐producing isolates. Branch colors indicate species‐level assignments. Outer ring: BLAST query coverage (50%–100%). Bootstrap values (1000 replicates) shown at major nodes. (B) Radar plot of species distribution. (C) Representative γ‐hemolysis (non‐hemolytic) phenotype on sheep blood agar. (D) Representative α‐hemolysis phenotype.

**Figure S3:** Detailed fecal metabolome characterization. (A, B) Chemical superclass composition of detected metabolites in (A) positive and (B) negative ion modes. (C, D) Top 15 differential metabolites by VIP score in (C) positive and (D) negative modes.

**Figure S4:** KEGG pathway enrichment of altitude‐associated differential metabolites and within‐pathway metabolite localization. (A) KEGG pathway enrichment analysis: Sankey diagram showing metabolite–pathway attribution (left, ribbon width proportional to metabolite count) and enrichment‐score plot of the four significantly enriched pathways (FDR < 0.05): sphingolipid metabolism, arachidonic acid metabolism, tryptophan metabolism, and steroid hormone biosynthesis. (B–E) Localization of upregulated differential metabolites within each enriched KEGG pathway: (B) arachidonic acid metabolism (ko00590), (C) steroid hormone biosynthesis (ko00140), (D) sphingolipid metabolism (ko00600), and (E) tryptophan metabolism (ko00380; kynurenine pathway branch‐specific regulation). Boxed values indicate log_2_ fold changes; all enriched metabolites were upregulated at high altitude.

**Table S1:** Per‐sample sequencing read processing statistics for 75 captive forest musk deer gut metagenomes.

**Table S2:** Species‐level relative‐abundance matrix for all 10,805 species across the 75 samples (Sheet 1 of 2). Differentially abundant species identified by LEfSe (71 species; LDA > 2, FDR < 0.05) (Sheet 2 of 2).

**Table S3:** MetaCyc pathway relative‐abundance matrix for all 533 pathways across the 75 samples (Sheet 1 of 2). Differentially abundant MetaCyc pathways identified by LEfSe (49 pathways; LDA > 2, FDR < 0.05) (Sheet 2 of 2).

**Table S4:** Complete catalog of all 6751 annotated fecal metabolites (Sheet 1 of 2). Altitude‐associated differential metabolites with VIP scores, log_2_ fold changes, FDR values, and KEGG pathway annotations (1682 metabolites; Sheet 2 of 2).

## Data Availability

Raw shotgun metagenomic sequencing reads for all 75 samples have been deposited in the NCBI Sequence Read Archive under BioProject accession PRJNA1442370. Untargeted fecal metabolomics data have been deposited in the MetaboLights repository (Yurekten et al. 2024) under study identifier MTBLS14151 (https://www.ebi.ac.uk/metabolights/MTBLS14151). Per‐sample read processing statistics are provided in Table S1. Analysis scripts for all components of this study have been organized into a dedicated GitHub repository (https://github.com/Berling996/musk‐deer‐altitude‐microbiome) and archived with the permanent Zenodo DOI https://doi.org/10.5281/zenodo.19805779.
