# Supplementary figures and images for: Altitude‐Associated Divergence of the Gut Microbiome in Endangered Forest Musk Deer: Evidence From Integrated Metagenomics, Metabolomics, and Culturomics
Source: Evol Appl. 2026 Jun 22;19(6):e70285. doi: 10.1111/eva.70285 (PMC13287323; doi:10.1111/eva.70285)

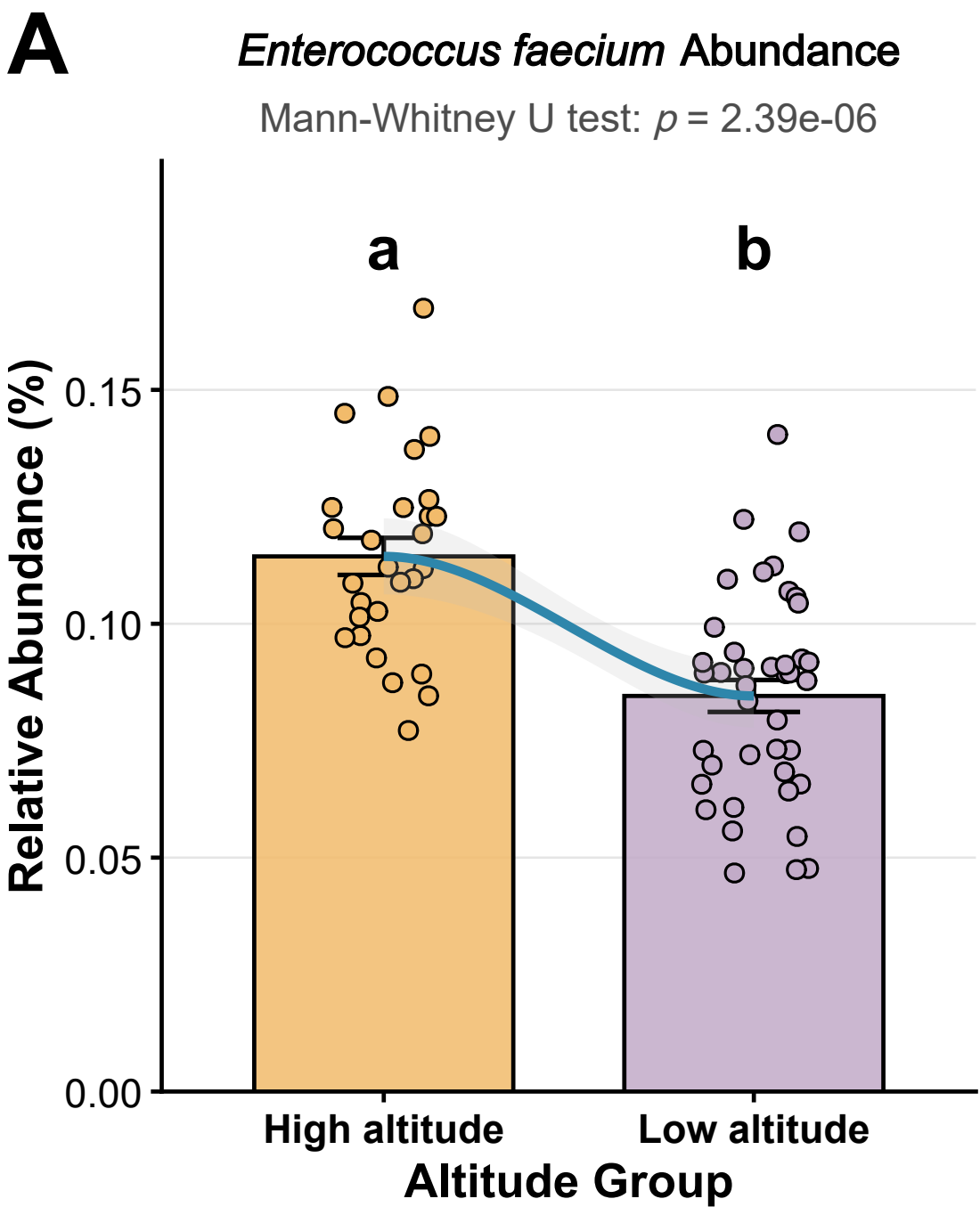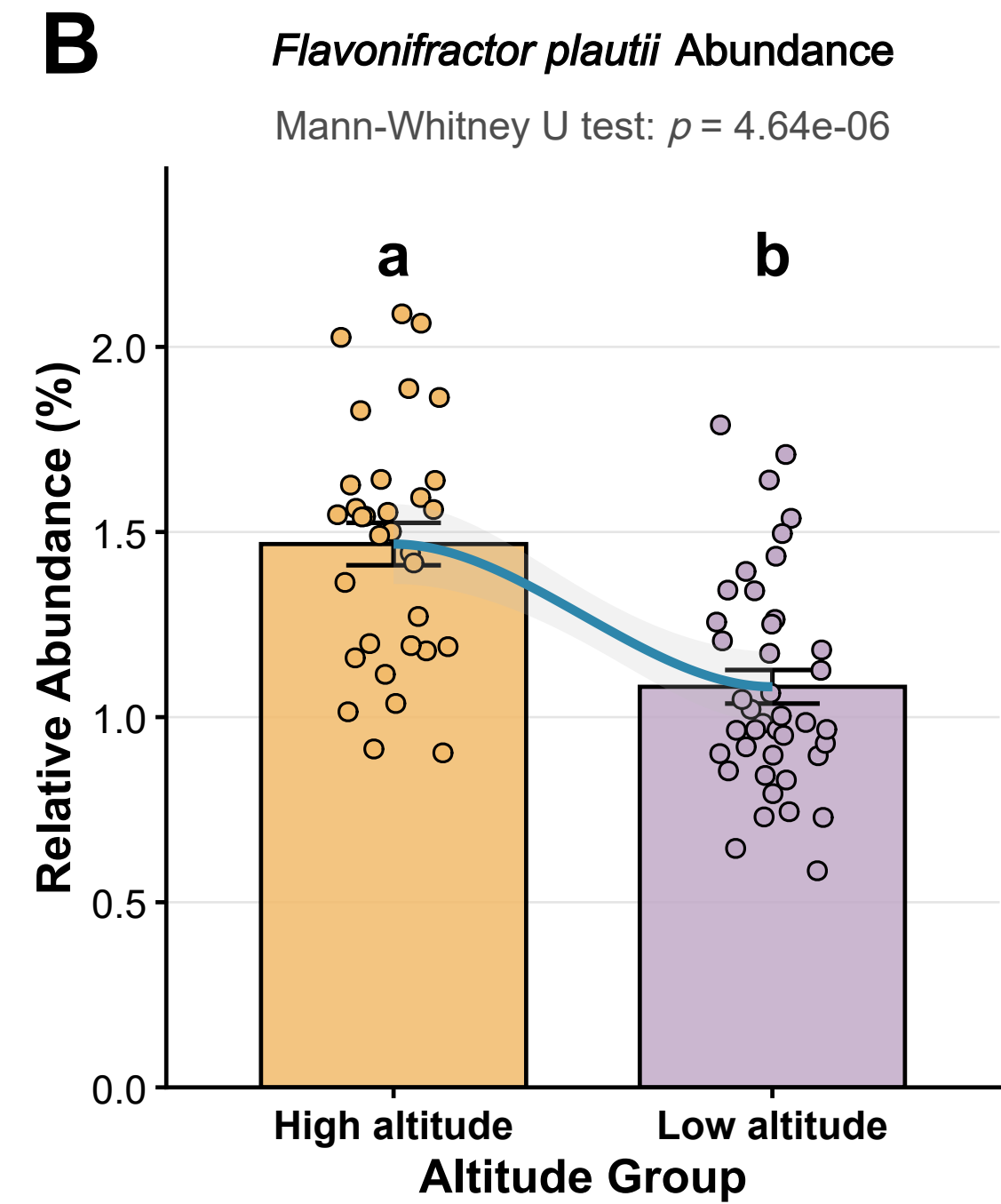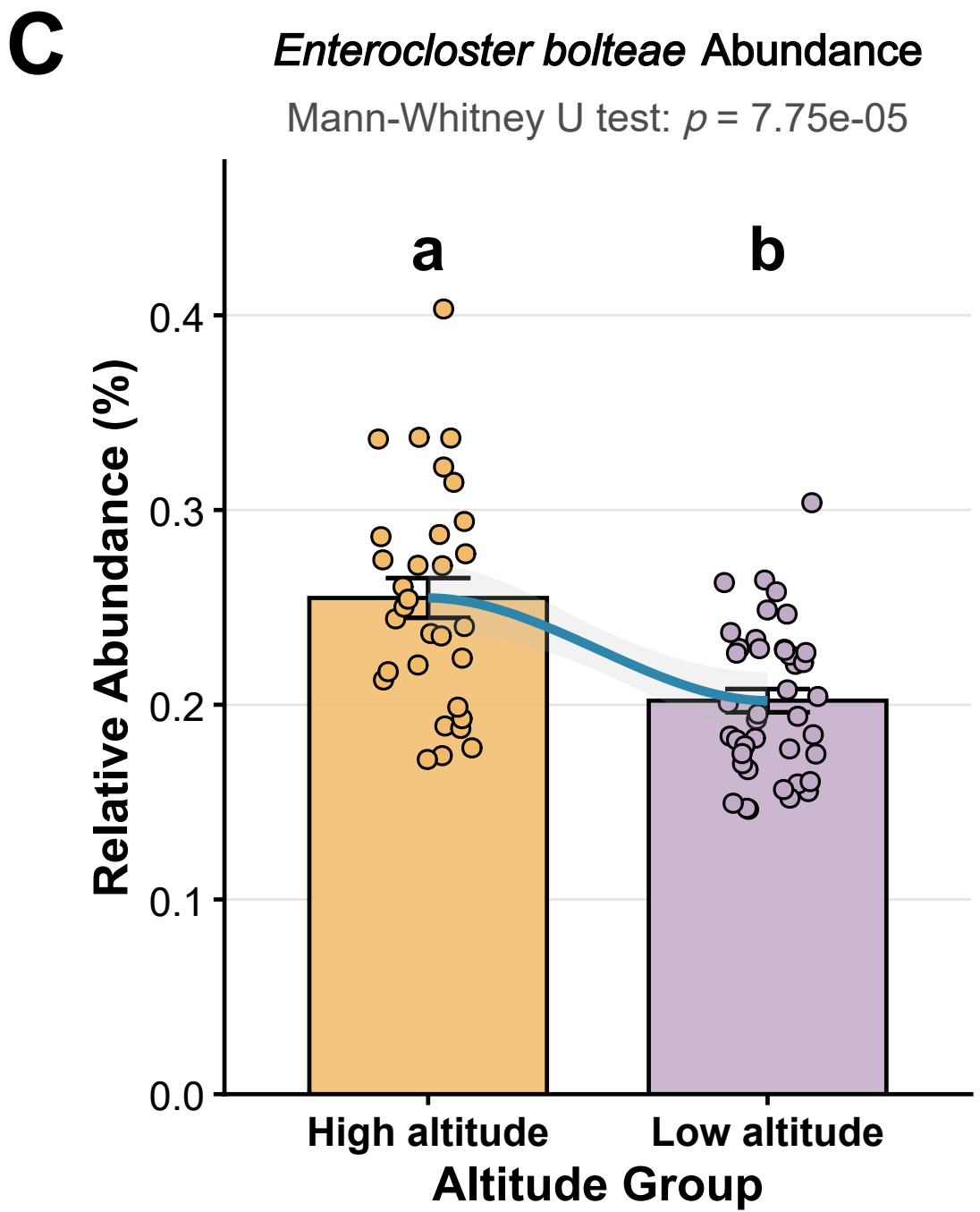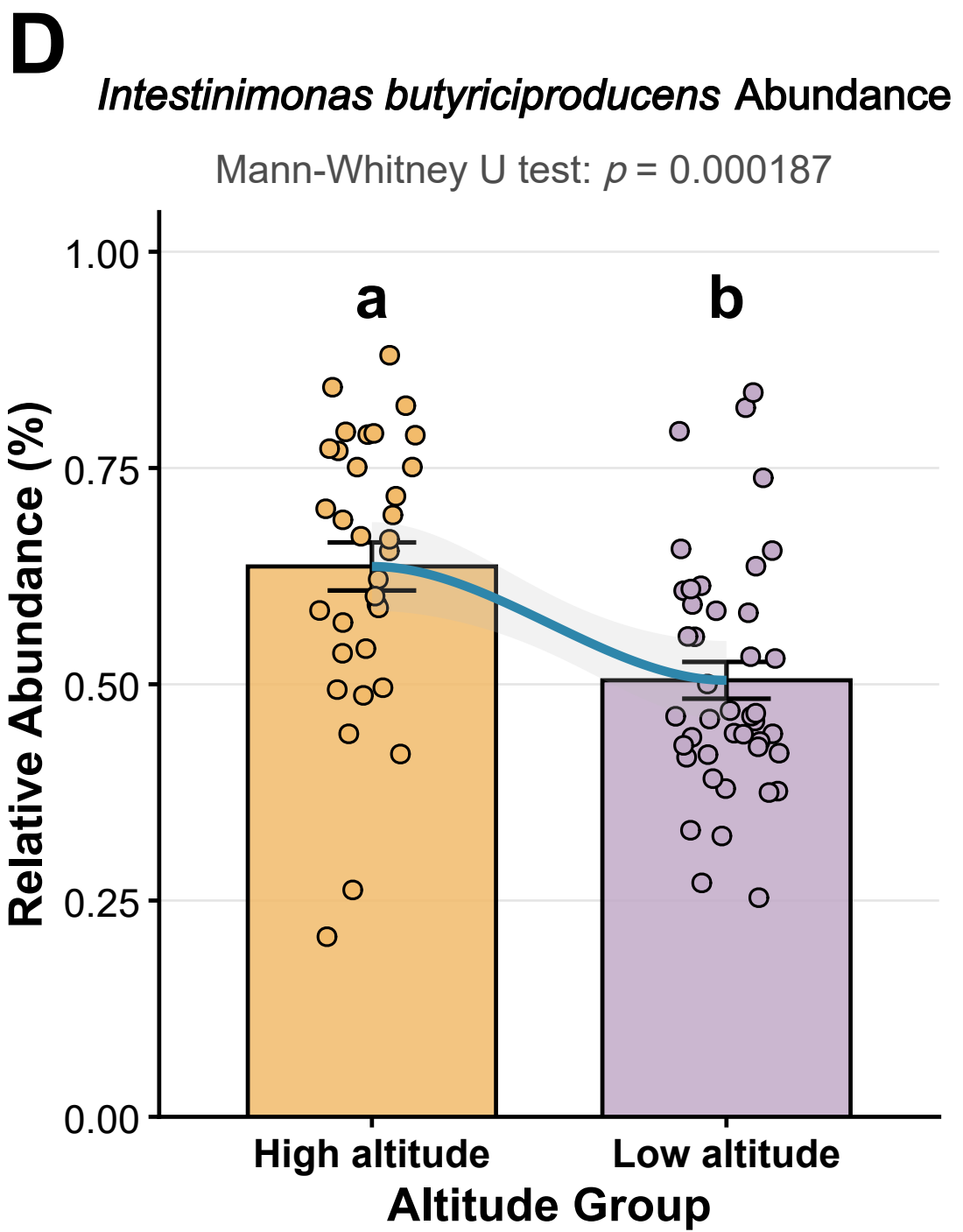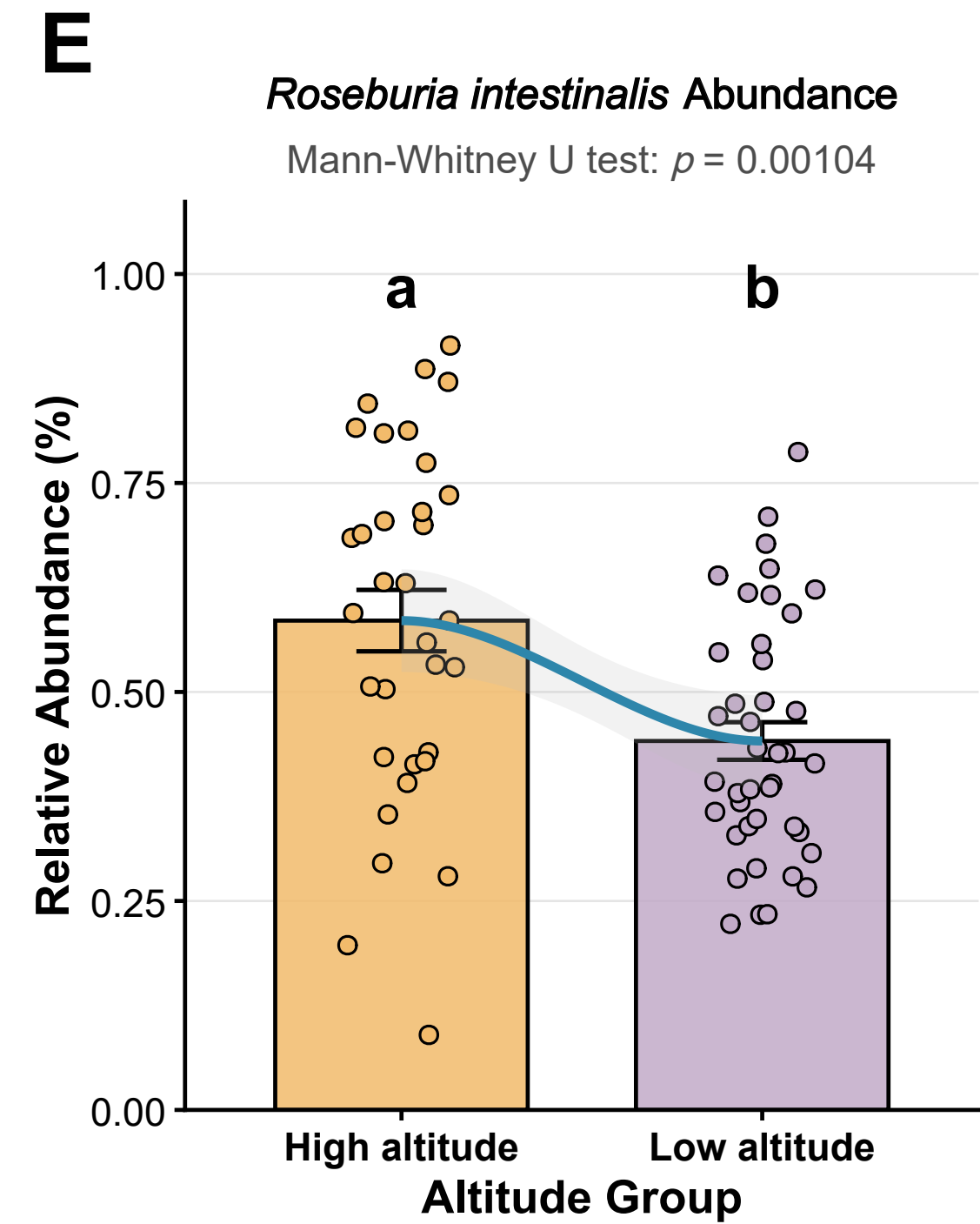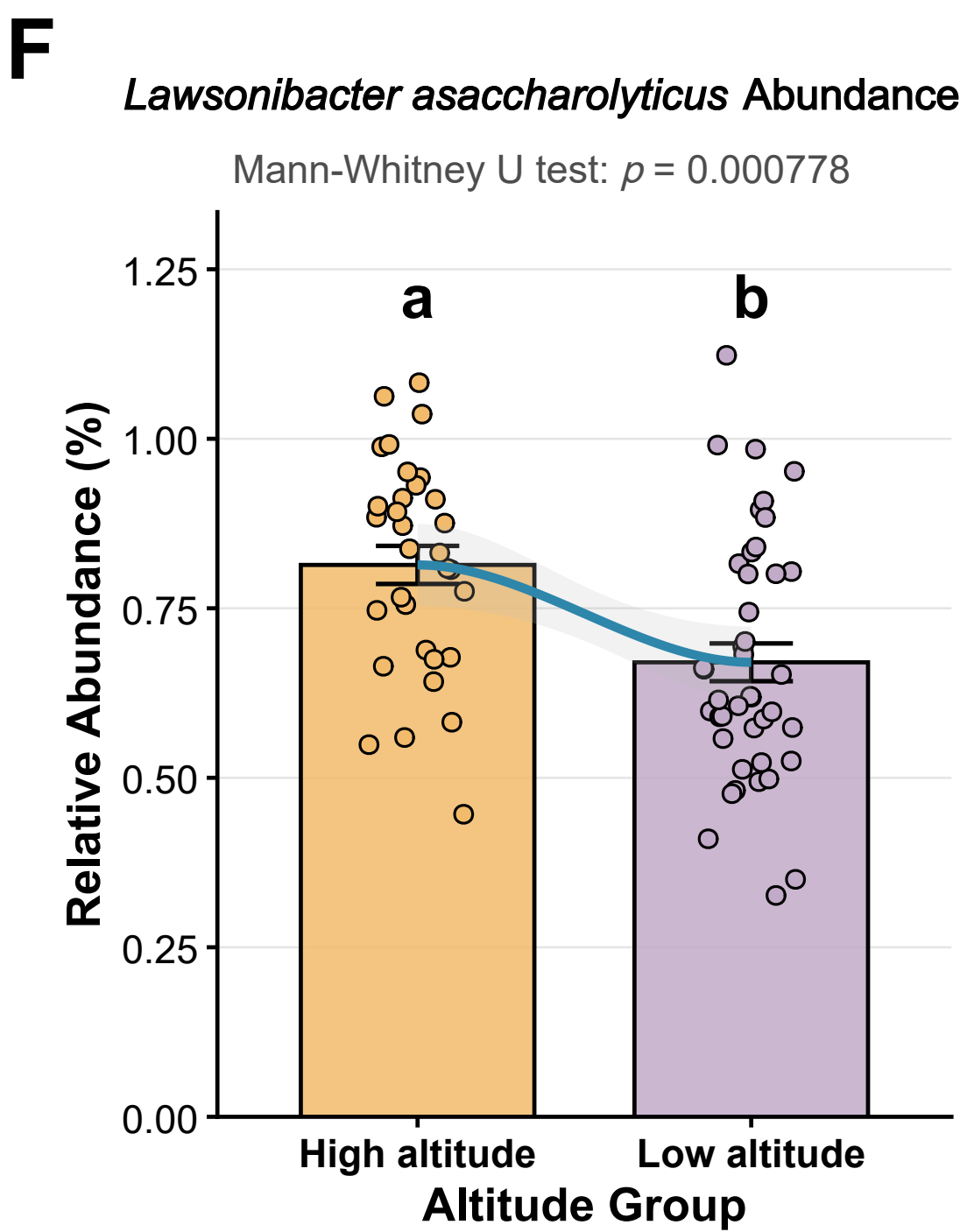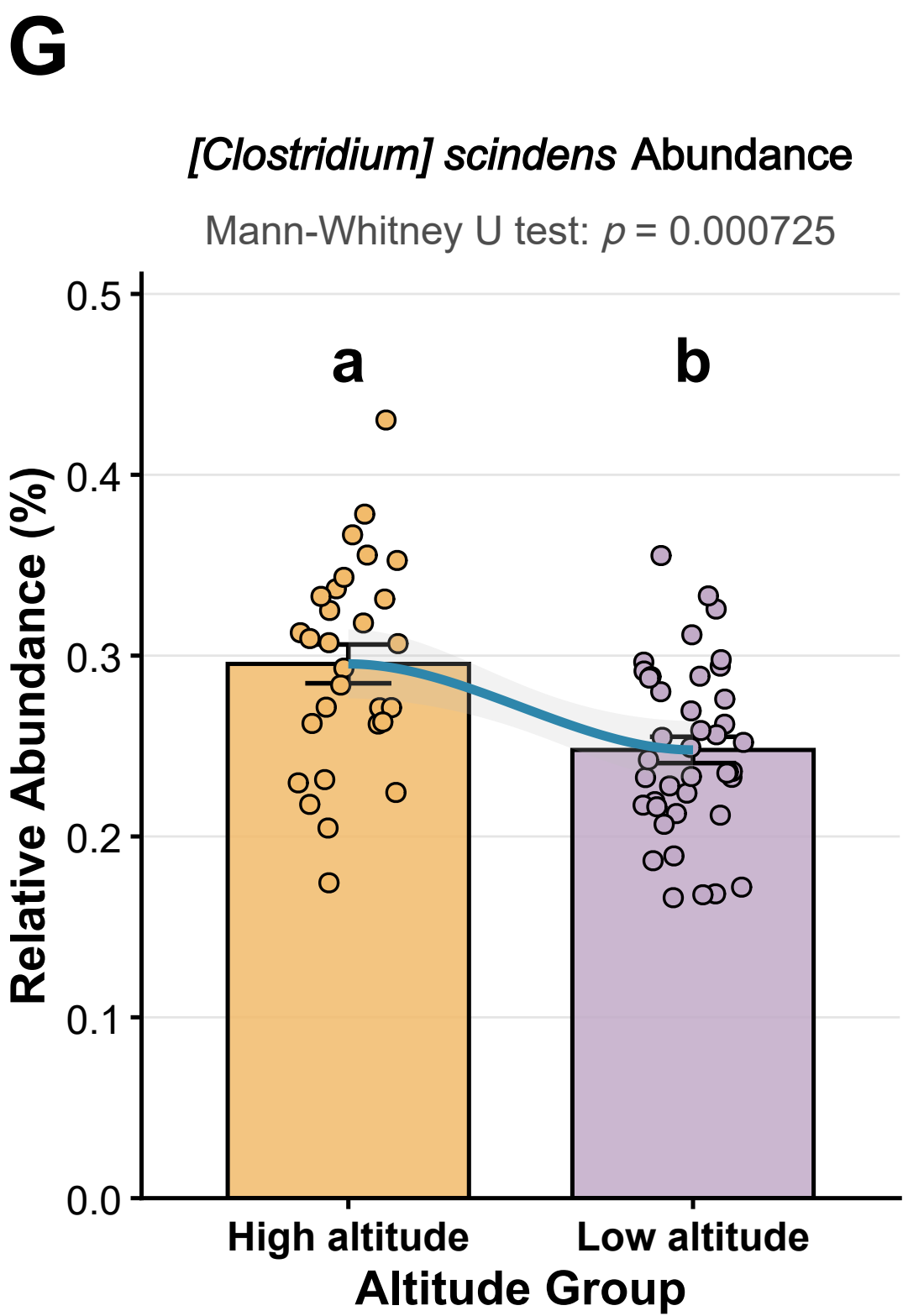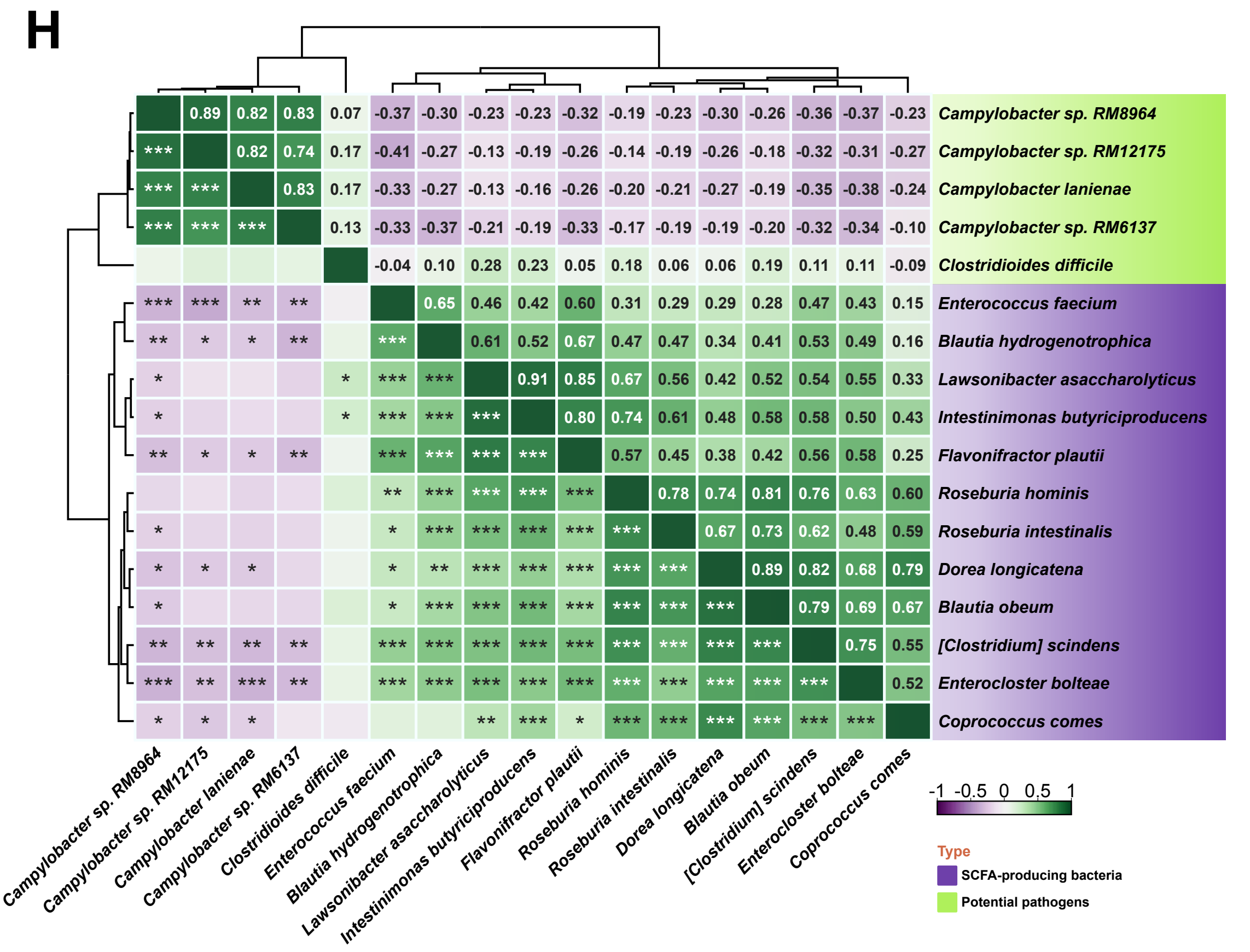

Supplement: Supplementary file 1 — Figure S1: Enrichment of SCFA‐producing bacteria and species correlation network. (A–G) Relative abundance of key SCFA‐producing bacteria between altitude groups: (A) Enterococcus faecium , (B) Flavonifractor plautii , (C) Enterocloster bolteae, (D) Intestinimonas butyriciproducens, (E) Roseburia intestinalis , (F) Lawsonibacter asaccharolyticus, (G) [Clostridium] scindens. Bars: means ± SEM; circles: individual values. Orange: high altitude; purple: low altitude. (H) Spearman correlation heatmap with hierarchical clustering among 17 differentially abundant species. [file EVA-19-e70285-s006.pdf]

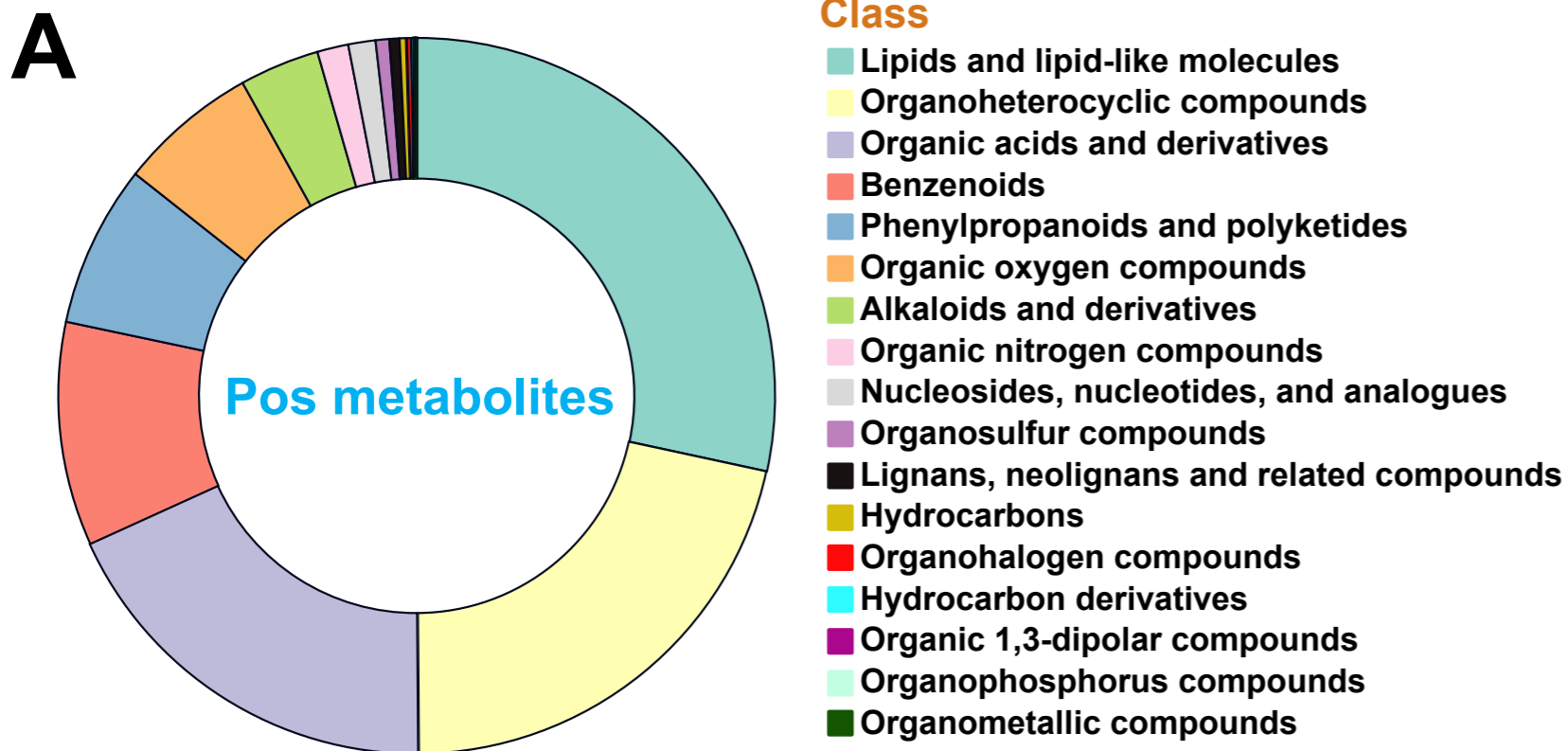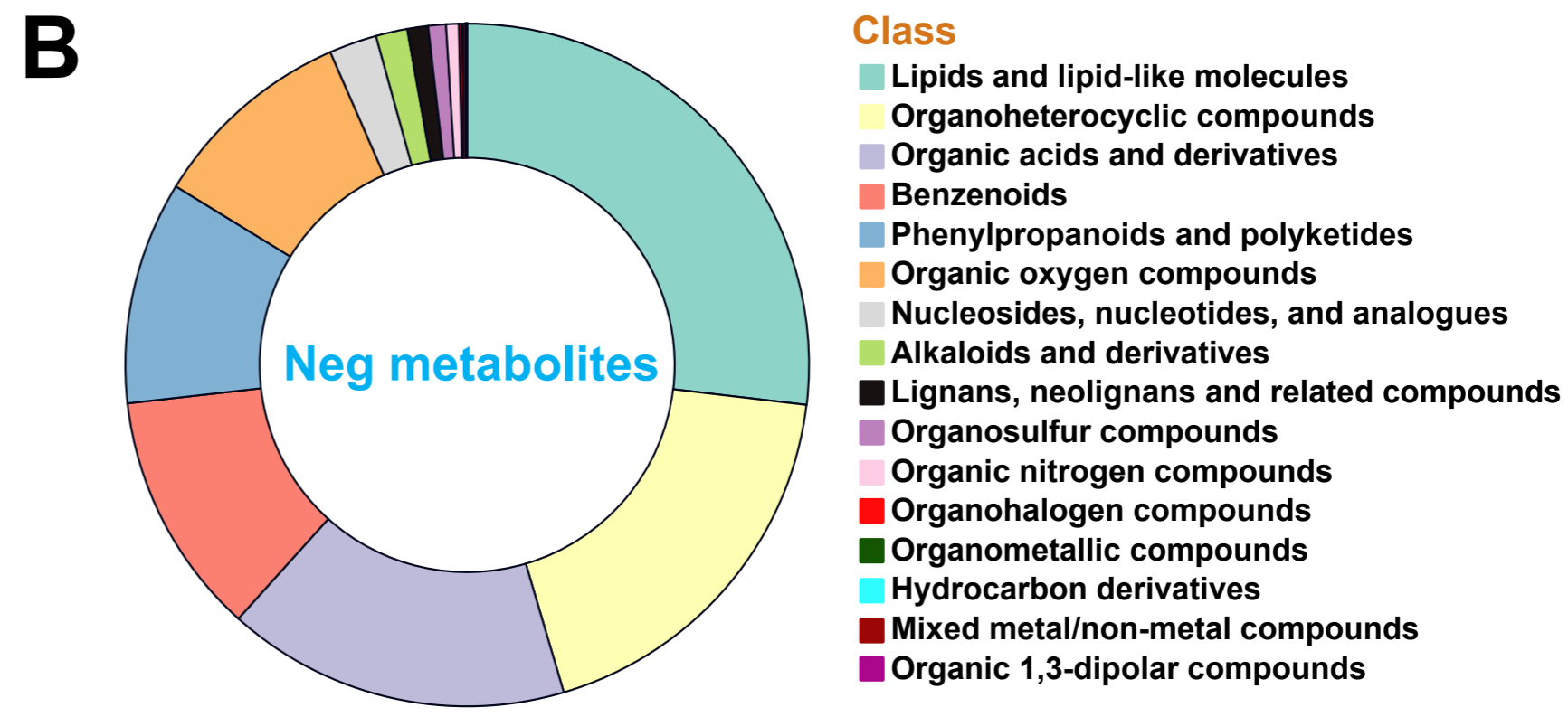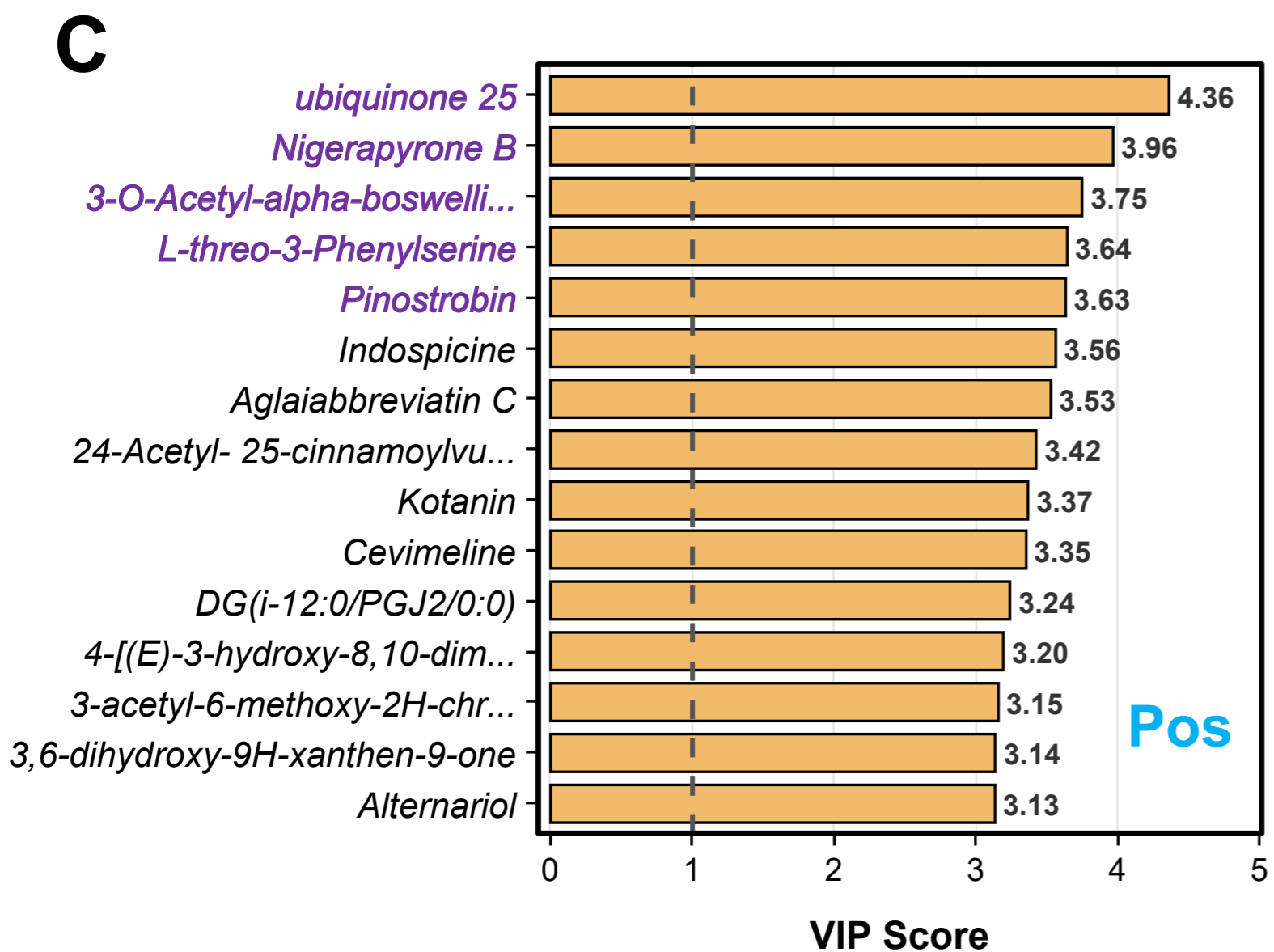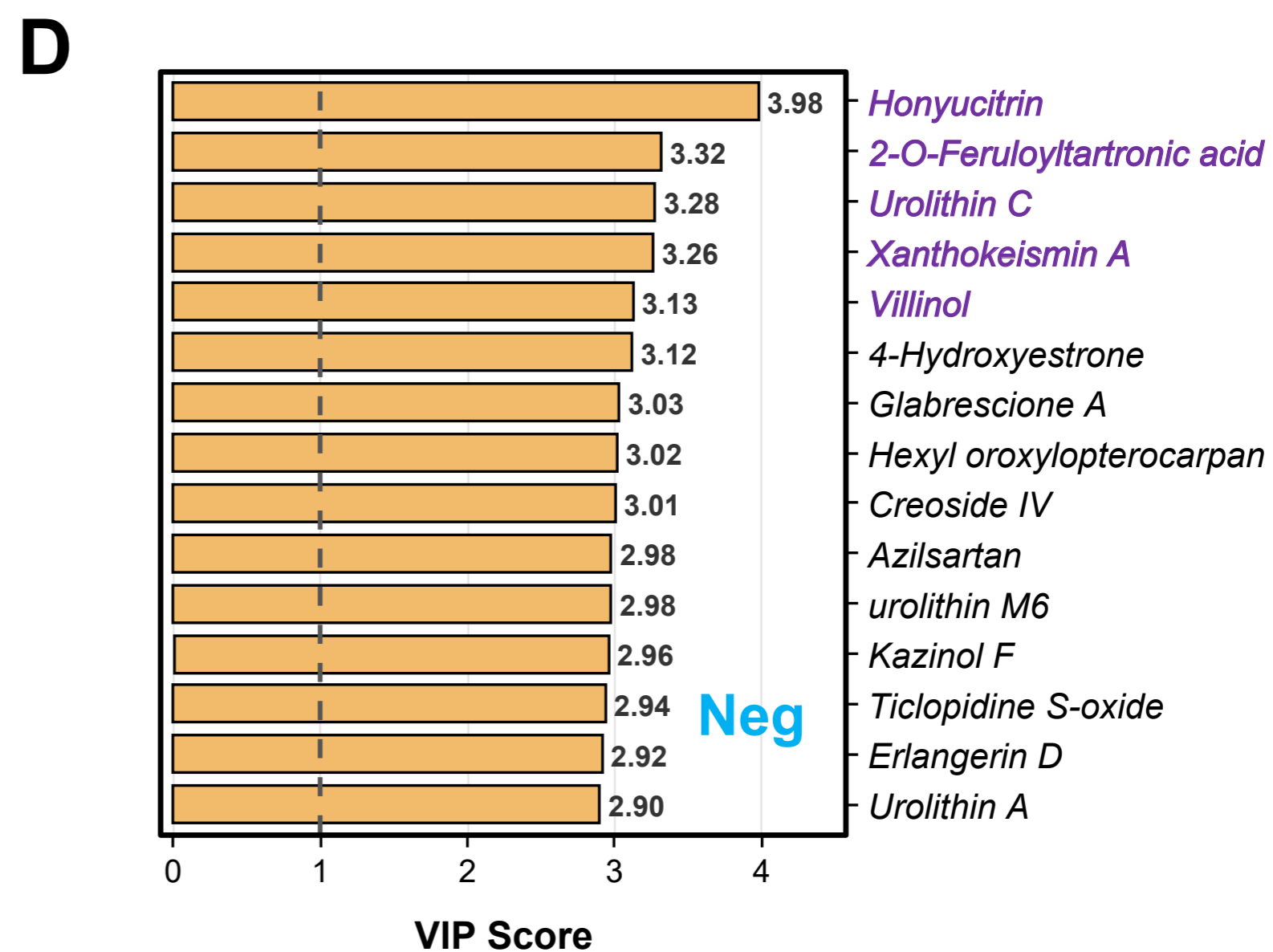

Supplement: Supplementary file 3 — Figure S3: Detailed fecal metabolome characterization. (A, B) Chemical superclass composition of detected metabolites in (A) positive and (B) negative ion modes. (C, D) Top 15 differential metabolites by VIP score in (C) positive and (D) negative modes. [file EVA-19-e70285-s008.pdf]

A

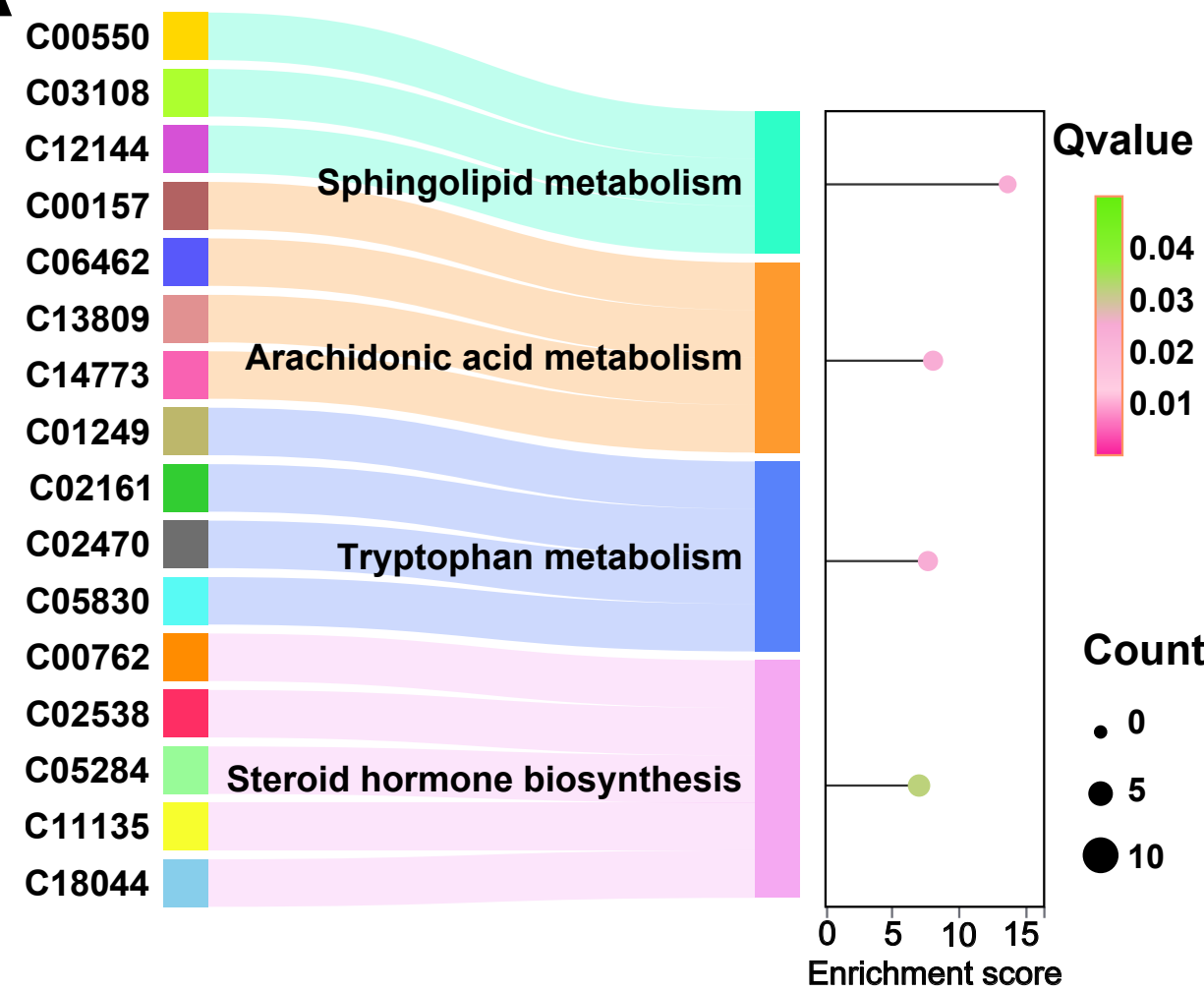

B

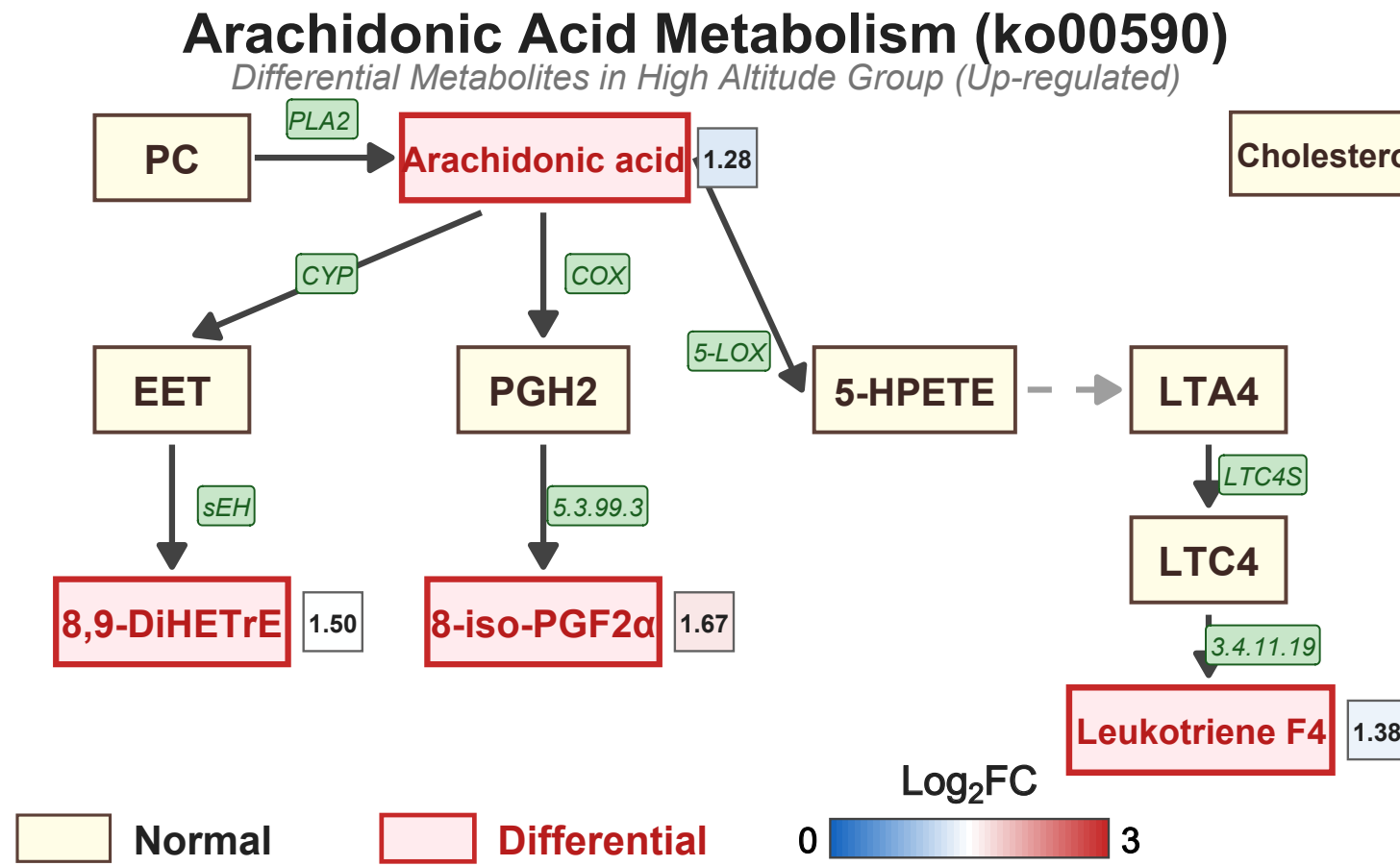

C

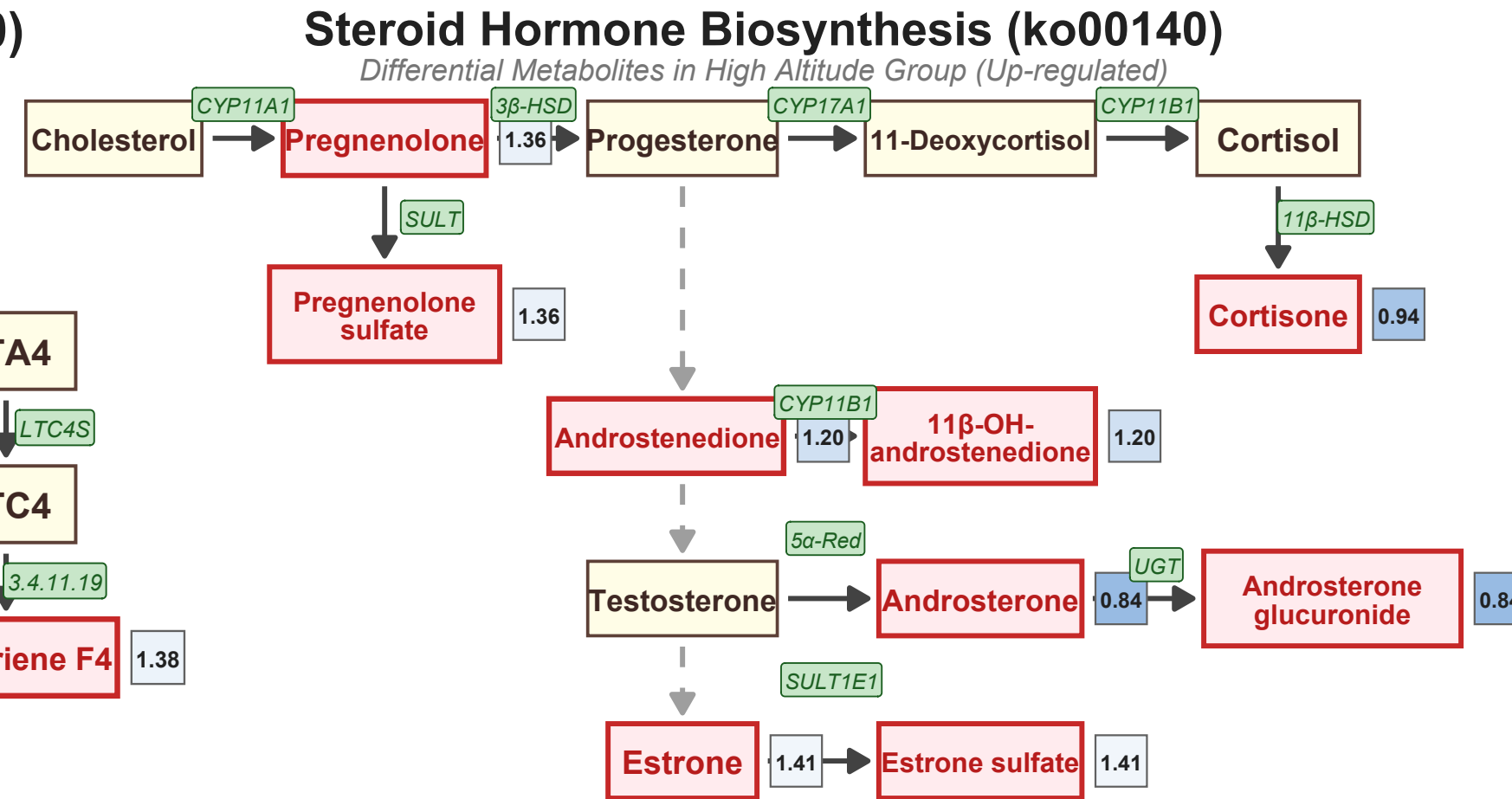

D

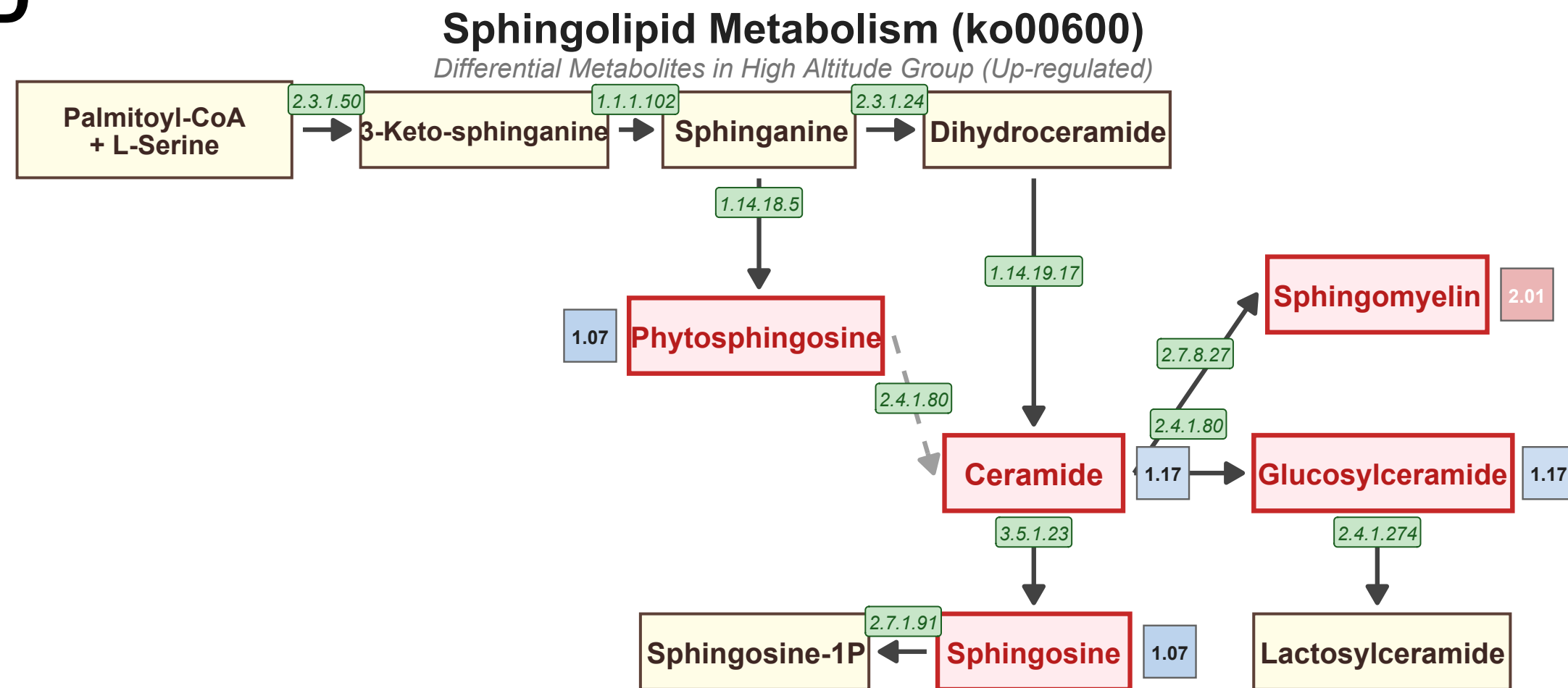

E

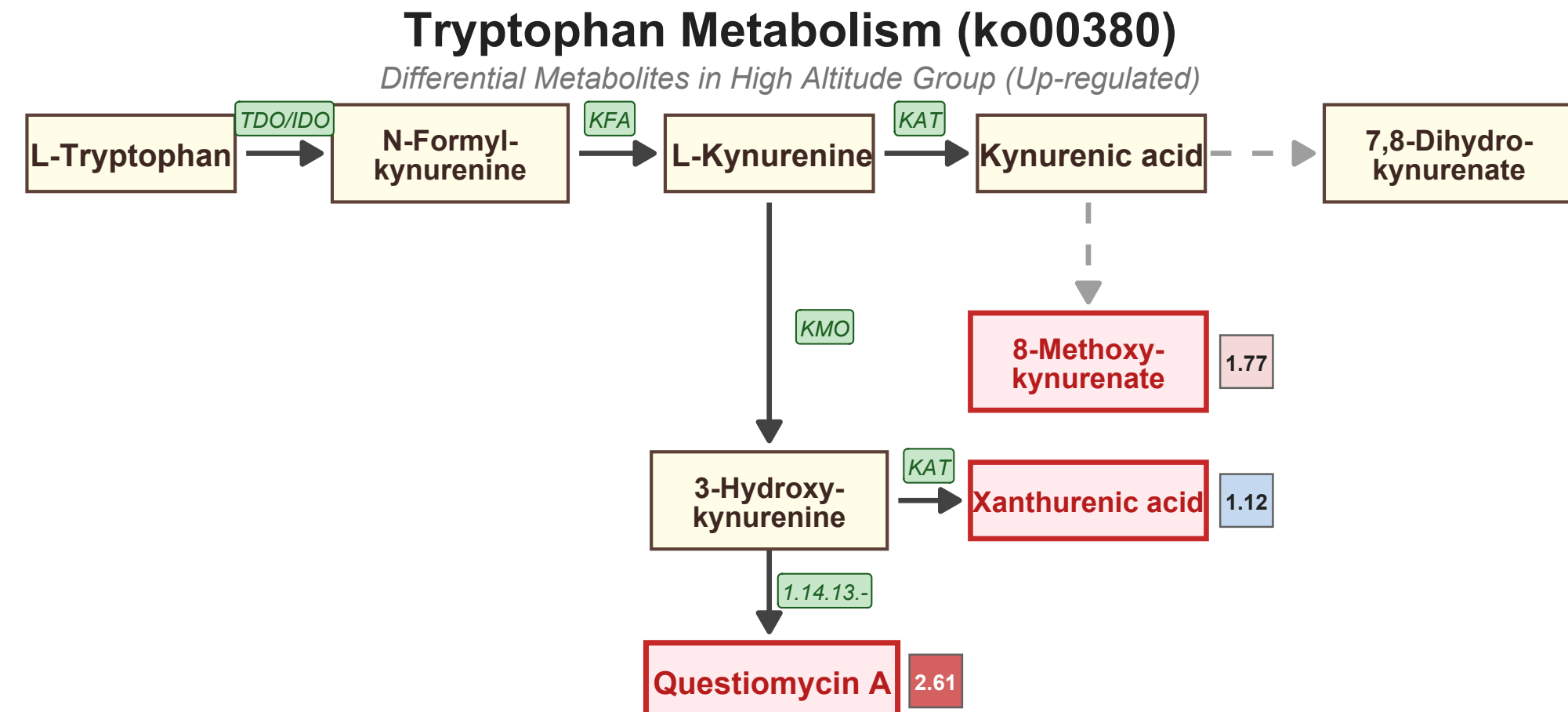

Supplement: Supplementary file 4 — Figure S4: KEGG pathway enrichment of altitude‐associated differential metabolites and within‐pathway metabolite localization. (A) KEGG pathway enrichment analysis: Sankey diagram showing metabolite–pathway attribution (left, ribbon width proportional to metabolite count) and enrichment‐score plot of the four significantly enriched pathways (FDR < 0.05): sphingolipid metabolism, arachidonic acid metabolism, tryptophan metabolism, and steroid hormone biosynthesis. (B–E) Localization of upregulated differential metabolites within each enriched KEGG pathway: (B) arachidonic acid metabolism (ko00590), (C) steroid hormone biosynthesis (ko00140), (D) sphingolipid metabolism (ko00600), and (E) tryptophan metabolism (ko00380; kynurenine pathway branch‐specific regulation). Boxed values indicate log2 fold changes; all enriched metabolites were upregulated at high altitude. [file EVA-19-e70285-s002.pdf]
