# Supplementary material for: Altitude‐Associated Divergence of the Gut Microbiome in Endangered Forest Musk Deer: Evidence From Integrated Metagenomics, Metabolomics, and Culturomics
Source: Evol Appl. 2026 Jun 22;19(6):e70285. doi: 10.1111/eva.70285 (PMC13287323; doi:10.1111/eva.70285)
Supplement: Supplementary file 2 — Figure S2: Phylogenetic identification and hemolytic activity screening of acid‐producing isolates. (A) Neighbor‐joining phylogenetic tree (16S rRNA) of 112 acid‐producing isolates. Branch colors indicate species‐level assignments. Outer ring: BLAST query coverage (50%–100%). Bootstrap values (1000 replicates) shown at major nodes. (B) Radar plot of species distribution. (C) Representative γ‐hemolysis (non‐hemolytic) phenotype on sheep blood agar. (D) Representative α‐hemolysis phenotype. [file EVA-19-e70285-s004.pdf]

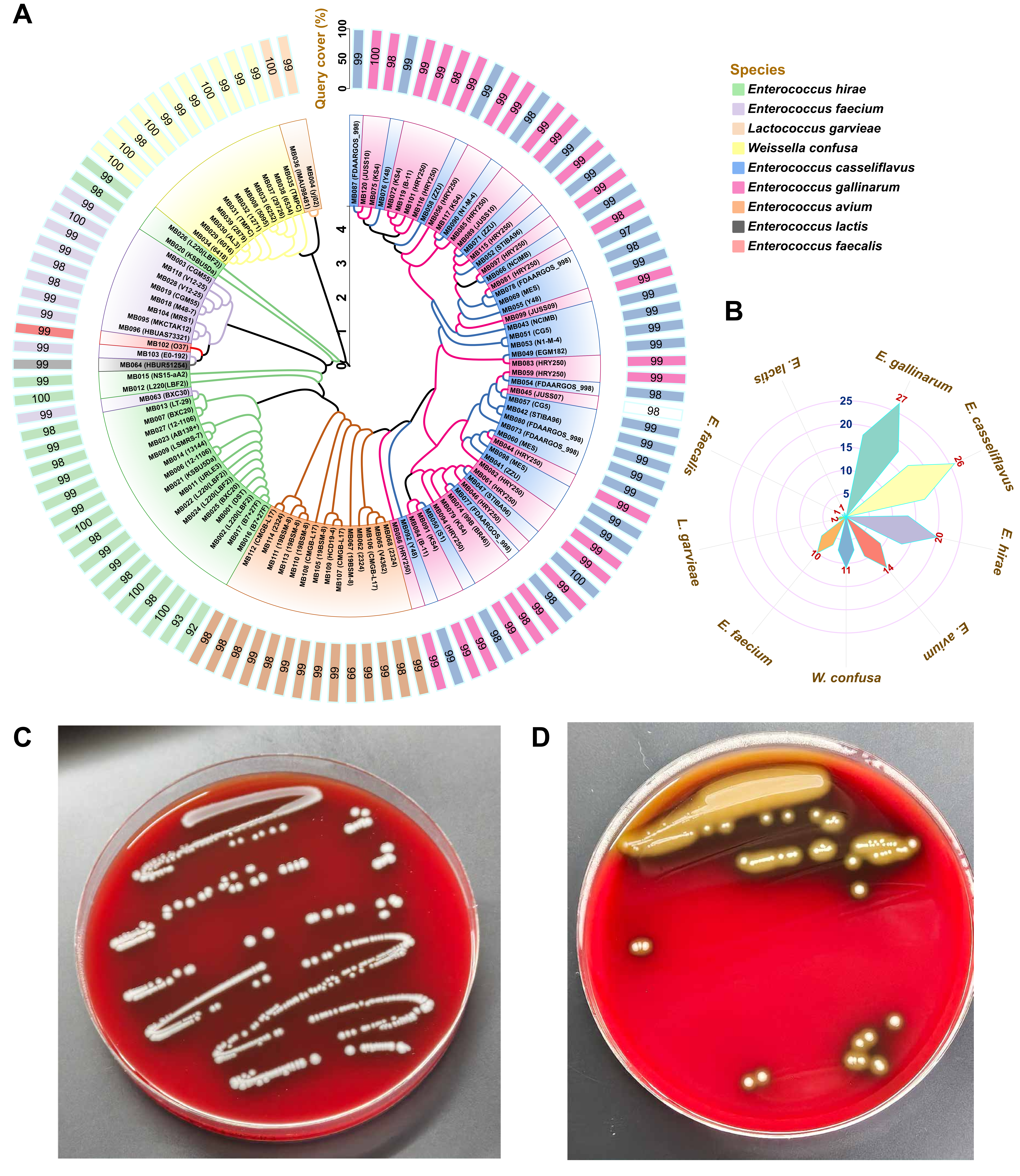

A
Query cover (%)
100
50
0
Species
Enterococcus hirae
Enterococcus faecium
Lactococcus garvieae
Weissella confusa
Enterococcus casseliflavus
Enterococcus gallinarum
Enterococcus avium
Enterococcus lactis
Enterococcus faecalis
B
E. gallinarum
E. casseliflavus
E. hirae
E. avium
W. confusa
E. faecium
L. garvieae
E. faecalis
E. lactis
25
20
15
10
5
27
26
20
14
11
10
2
1
1
C
D
